# A Comprehensive Map of Mobile Element Insertion Polymorphisms in Humans

**DOI:** 10.1371/journal.pgen.1002236

**Published:** 2011-08-18

**Authors:** Chip Stewart, Deniz Kural, Michael P. Strömberg, Jerilyn A. Walker, Miriam K. Konkel, Adrian M. Stütz, Alexander E. Urban, Fabian Grubert, Hugo Y. K. Lam, Wan-Ping Lee, Michele Busby, Amit R. Indap, Erik Garrison, Chad Huff, Jinchuan Xing, Michael P. Snyder, Lynn B. Jorde, Mark A. Batzer, Jan O. Korbel, Gabor T. Marth, 1000 Genomes Project

**Affiliations:** 1 Department of Biology, Boston College, Chestnut Hill, Massachusetts, United States of America; 2 Department of Biological Sciences, Louisiana State University, Baton Rouge, Louisiana, United States of America; 3 Genome Biology Unit, European Molecular Biology Laboratory, Heidelberg, Germany; 4 Department of Genetics, Stanford University, Stanford, California, United States of America; 5 Department of Human Genetics, Eccles Institute of Human Genetics, University of Utah, Salt Lake City, Utah, United States of America; Fred Hutchinson Cancer Research Center, United States of America

## Abstract

As a consequence of the accumulation of insertion events over evolutionary time, mobile elements now comprise nearly half of the human genome. The Alu, L1, and SVA mobile element families are still duplicating, generating variation between individual genomes. Mobile element insertions (MEI) have been identified as causes for genetic diseases, including hemophilia, neurofibromatosis, and various cancers. Here we present a comprehensive map of 7,380 MEI polymorphisms from the 1000 Genomes Project whole-genome sequencing data of 185 samples in three major populations detected with two detection methods. This catalog enables us to systematically study mutation rates, population segregation, genomic distribution, and functional properties of MEI polymorphisms and to compare MEI to SNP variation from the same individuals. Population allele frequencies of MEI and SNPs are described, broadly, by the same neutral ancestral processes despite vastly different mutation mechanisms and rates, except in coding regions where MEI are virtually absent, presumably due to strong negative selection. A direct comparison of MEI and SNP diversity levels suggests a differential mobile element insertion rate among populations.

## Author Summary

We embarked on this study to explore the 1000 Genomes Project (1000GP) pilot dataset as a substrate for Mobile Element Insertion (MEI) discovery and analysis. MEI is already well known as a significant component of genetic variation in the human population. However the full extent and effects of MEI can only be assessed by accurate detection in large whole-genome sequencing efforts such as the 1000GP. In this study we identified 7,380 distinct genomic locations of variant MEI and carried out rigorous validation experiments that confirmed the high accuracy of the detected events. We were able to measure the frequency of each variant in three continental population groups and found that inherited MEI variants propagate through populations in much the same way as single nucleotide polymorphisms, except that MEI are more strongly suppressed in protein coding parts of the genome. We also found evidence that the MEI mutation rate has not been constant over human population history, rather that different populations appear to have different characteristic MEI mutation rates.

## Introduction

### Mobile elements: significance and current catalogs

Retrotransposons are endogenous genomic sequences that copy and paste into locations throughout host genomes [1]–[3]. Most mobile elements annotated in the human reference genome are remnants of ancient retrotransposition events and are no longer capable of active retrotransposition. However, a fraction of mobile elements remain active and contribute to variation between individuals in the human population. These active elements belong almost exclusively to the Alu, L1, and SVA families of non-LTR retrotransposons [4].

The Alu family is the most common mobile element in primate genomes, with more than 1.1 million copies in *Homo sapiens* [5]–[7]. The sequence of a full-length Alu element is 300 bp long. Alu elements are classified into a range of sub-families which have different propensities for retrotransposition, and are identified according to sequence alterations. Several AluY sub-families are currently active and are responsible for the bulk of mobile element insertion variation in *Homo sapiens*. The human reference genome contains over 140,000 annotated AluY elements. After Alus, L1 insertions are the next most prevalent family of mobile elements. There are over 500,000 L1 elements annotated in *Homo sapiens*. A full-length L1 is a sequence of roughly 6 kb in length and the most active L1 sub-family in the human lineage is L1HS [8], [9]. There are a little more than 1,500 L1HS annotated elements in the human reference. A third family of mobile element are SVA retrotransposons [10]. SVAs are hybrid elements of SINE, VNTR and Alu components that range in size up to several Kb, with more than 3,600 annotated SVA elements in the human reference genome. SVA elements are thought to be the youngest family of retrotransposons in primates [11]. Other less common classes of mobile elements, such as DNA transposons, and endogenous retroviruses are not the focus in this study.

Mobile element insertions (MEI) are known to generate significant structural variation within *Homo sapiens* [12], [13] and have diverse functional impacts [14]–[16]. In vitro experiments identified key features of Alu [17] and L1 [18] elements responsible for retrotransposon activity. The identification of MEI variant loci in humans initially began with disease-causing insertion events (e.g. hemophilia [19], breast cancer [20]). Experimental approaches were based upon library screening and small-scale PCR based display assays [21]. These approaches have been augmented by comparisons of the NCBI and the HuRef genomes [22], [23], large scale fosmid-end sequences [24], and targeted sequencing of element-specific PCR products [25]–[28]. The dbRIP database of MEI polymorphisms [29] currently contains 2,691 polymorphic loci, enabling early estimates for the total number of segregating events [25] and per-generation mutation rates [23].

MEI polymorphisms can be detected either as insertions or as deletions in samples relative to the reference genome. Mechanistically, however, both types of observations are due to retrotransposon insertion; precise excisions of mobile elements are essentially non-existent [1]. Therefore MEI detected as deletions are, in fact, retrotransposon insertions in the reference DNA and can be verified as such by comparison with ancestral genomes. Detection and genotyping properties of MEI detected as insertions (“*non-reference MEI*”) and as deletions (“*reference MEI*”) are substantially different. We present their respective properties separately before combining the two detection modes into a unified MEI analysis. The deletion detection methods and properties of the full set of 1000GP deletions have been extensively described in the 1000GP CNV companion paper [30]. This allows us to focus on specific properties of the reference MEI subset of those deletions. Effective computational algorithms using second-generation sequencing data exist for identifying deletions [27], [31], [32], and have been used to find MEI in particular [33]. Detecting non-reference MEI directly as insertions from whole genome shotgun sequence data poses a more challenging problem, owing to the inherent difficulties associated with accurate mapping of sequenced reads derived from highly repetitive regions of the genome. Only recently have methods been developed for the purpose of non-reference MEI detection from second-generation whole genome shotgun data including published studies of L1 element insertions [34] and of Alu insertions [35]. These studies adopted similar computational approaches to one of our insertion detection methods (the read pair method, see Materials and Methods) and have different detection properties (Text S2 Comparisons, Figures S8, S9, S10).

Relative to previous studies, we present a broad analysis of MEI variation in the human population; with more variant loci detected, from the three major mobile element families, using multiple detection methods, each with comprehensive experimental validation (Table 1). The present study represents the combined efforts of the MEI sub-group of the 1000 Genomes Project and has been prepared as a companion to previous 1000GP pilot publications [30], [36]. The MEI analyzed in this study were included the 1000GP variant call release of July 2010 (ftp://ftp-trace.ncbi.nih.gov/1000genomes/ftp/pilot_data/release/2010_07), also provided as Table S1). The specific purpose here is to provide a more detailed description of the methods, validation experiments, and properties of the 1000GP catalog of MEI events, and to extend the analysis by adding genotype information, population allele frequencies, and population specific mutation rates.

**Table 1:** 1000 Genomes Project pilot data used for mobile element insertion discovery.

	Non-reference MEI					Reference MEI		
Detection method	Illumina RP		454 SR		RP+SR	Combined deletion detection algorithms		
Dataset	Low Cov	Trio	Low Cov	Trio	Total	Low Cov	Trio	Total
Number of samples	138	6	22	2	156	169	6	175
Coverage per sample	2.2x	16.4x	2.0x	7.6x	3.0x	3x	25x	3.9x
Alu insertions	2882	1786	2420	1284	4500	1689	1420	1730
L1 insertions	345	192	396	172	792	193	170	206
SVA insertions	49	35	17	7	79	70	65	74
Loci PCR tested	193	186	182	185	746	-	-	-
Loci validated	183	182	173	174	712	1873	1615	1927
FDR (%)	5.2±1.6	2.2±1.1	4.4±1.6	5.5±1.1	4.5±0.8	-	-	-

Number of samples, average read coverage, detected loci, and validation results are shown. Non-reference MEI false detection rates (FDR) were based on validation results at randomly selected loci. In addition to PCR validation, reference MEI were also tested for validation as deletions by local assembly. The FDR for reference MEI, including the additional MEI selection criteria, is estimated to be <10%.

## Results

### Datasets analyzed

We analyzed two whole-genome datasets produced by the 1000GP, the low coverage pilot dataset consisting of 179 individuals sequenced to ∼1–3X coverage and the trio pilot dataset consisting of two family trios sequenced to high, ∼15–40X coverage (Table S2, Figure S4). These datasets included samples from three continental population groups, 60 samples of European origin (CEU), 59 African (YRI), and 60 Asian samples from Japan and China (CHBJPT). The two pilot datasets were produced and analyzed for complementary purposes. The trio dataset was used for assessing detection methods in high coverage samples and for the purpose of finding candidate *de novo* insertions in the trio children. The high coverage dataset was used to assess population properties of MEI. Both datasets contributed to the overall catalog of events.

### Detection of non-reference mobile element insertions

**Figure 1:**
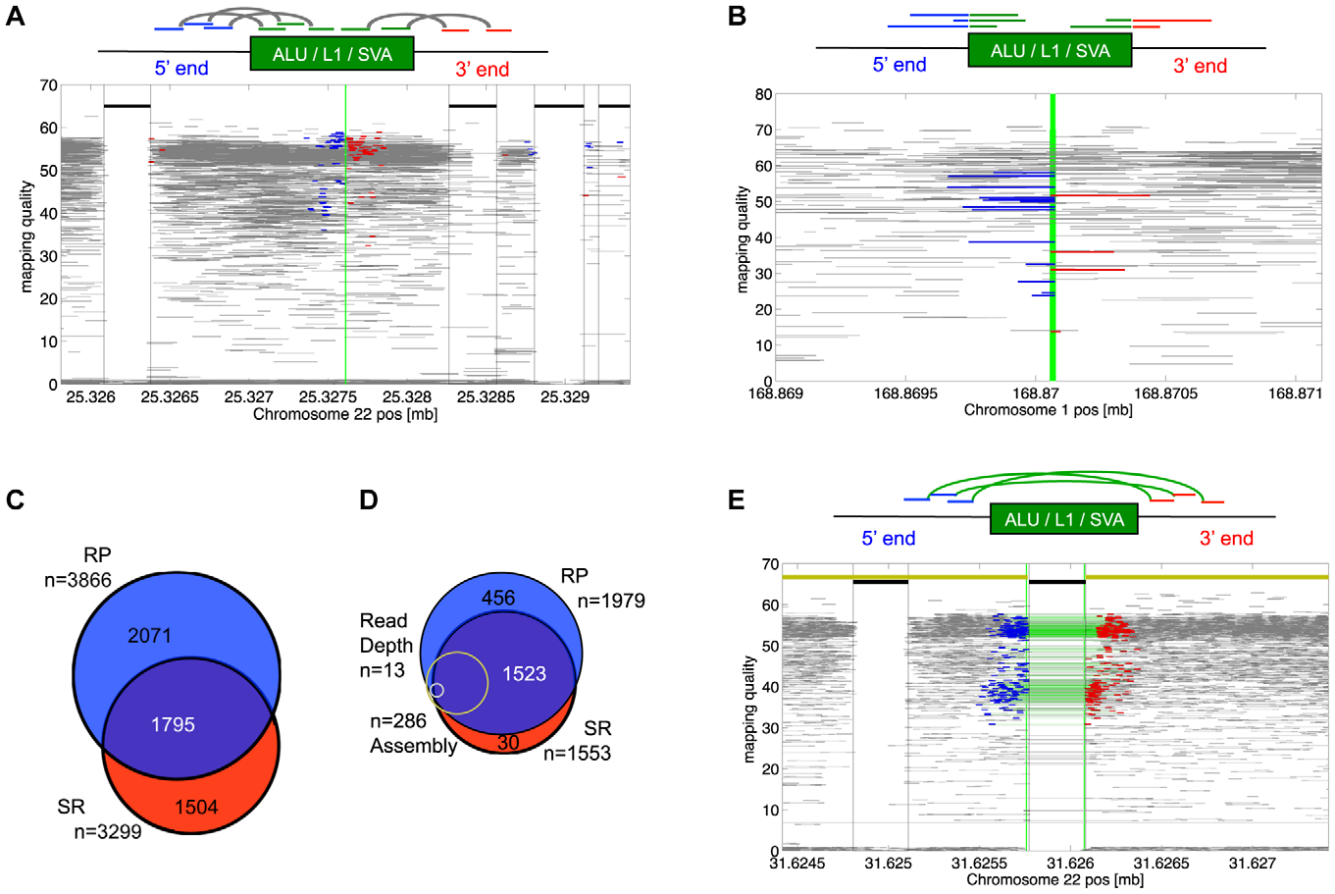
MEI detection modes. a) RP signature for of non-reference MEI detection. The RP signature consists of Illumina read pairs spanning into the element from each side of the insertion. The RP event display shows a heterozygous Alu insertion allele on chromosome 22 from the trio pilot dataset. Fragment mapping quality is shown on the vertical scale. Horizontal grey lines show read pairs uniquely mapped at both ends with a mapped fragment length consistent with the sequence library; the blue and red lines are read pairs spanning into an Alu sequence from the 5′ and 3′ ends. The green vertical line is the position of the insertion. Thick black lines near the top show annotated Alu positions. Red and blue reads bracketing annotated elements are characteristic of mapping artifacts that we removed from insertion detection by masking out regions within a fragment length of an annotated element of the same family as the insertion. b) Signature for SR-based insertion detection. Split-mapped 454 reads span into the element sequence. The SR event display shows split reads spanning into an Alu insertion from the 5′ (blue) or the 3′(red) sides. The vertical green line marks the insertion site. Fully mapped 454 reads are shown in gray. Gray reads that span the breakpoint correspond to the reference allele. Note that the mapping quality increases with the length of the split-mapped segment. The red and blue segments overlap by roughly 15 bp in the target site duplication region that brackets the MEI insertion. c) Overlap between non-reference MEI detected by RP and by SR. d) Overlap between detection methods for reference MEI. Of the 23 1000GP deletion call sets, 11 were RP and 4 were SR. Also shown are the relative proportions of events detected by assembly (yellow) and by read depth (gray) both of which had nearly 100% overlap with RP and SR calls. e) RP signature for reference MEI detection. Read pairs with abnormally long mapped fragment lengths (in green) span over an AluYb8 annotation. The event display shows RP evidence for a homozygous reference MEI in chromosome 22 from the trio dataset. The yellow line at the top marks homologous regions from the chimpanzee assembly, with a gap at the precise location of the variant MEI.

We developed two complementary methods for the detection of non-reference MEI, a read-pair constraint (RP) method applied to Illumina paired-end short read data, and a split-read (SR) method applied to the longer read data from Roche/454 pyrosequencing (Materials and Methods: non-reference MEI detection). Figure 1a and 1b shows the respective detection signatures and examples of event displays. Candidate MEI events were formed as clusters of supporting fragments. A limitation specific to RP detection arises from annotated elements within a characteristic read pair fragment length of candidate MEI (Figure 1a). Read pairs spanning from a uniquely mapped anchor into an annotated mobile element with a fragment length consistent with the given library fragment length distribution (Figure S5) are characteristic of the reference allele and are not evidence for non-reference MEI. These “background” read pairs occasionally have fragment lengths on the extreme tails of the library distribution and can potentially be misclassified as evidence for non-reference MEI. For this reason RP detection criteria required at least two supporting fragments spanning into the insertions from both sides of the insertion. We also masked insertion positions within a fragment length around each annotated element of the corresponding family from RP detection in order to achieve a low false detection rate. The SR method was not dependent on the fragment length distribution in the 454 data so these additional detection criteria were not required.

We applied the two methods to both 1000GP pilot datasets (Table 1) separately, yielding a total of 5,370 distinct genomic MEI loci, 33% of which were found by both SR and RP methods (Figure 1c). The overall level of detection overlap between SR and RP methods is limited by detection sensitivity and specificity (see below) and the number of samples sequenced by both 454 and by Illumina read pairs.

### Detection of reference MEI

In addition to the 5,370 non-reference MEI, we identified 2,010 reference MEI detected as deletions of mobile elements in samples. The reference MEI events were selected from the full release set of 1000GP pilot deletions (n = 22025) [30], [36] based on matching deletion coordinates to RepeatMasker 3.27 Alu, L1, and SVA annotations [6], and the requirement that the mobile element is absent in the chimpanzee genome [37] (6x *pan Trogodytes*-2.1 assembly) at the corresponding positions in hg18 (Materials and Methods: Reference MEI selection). Figure 1e shows an example event display of an AluYb8 reference MEI, detected as a deletion in the trio pilot data. All but one of the reference MEI were found by one or more of the RP or SR deletion detection algorithms that were part of the released 1000GP deletion call set [30], [38]–[42] with a small overlapping contribution from algorithms based on assembly or read depth methods [43], [44] (Figure 1d, Table S3).

### Combined MEI catalog

**Figure 2:**
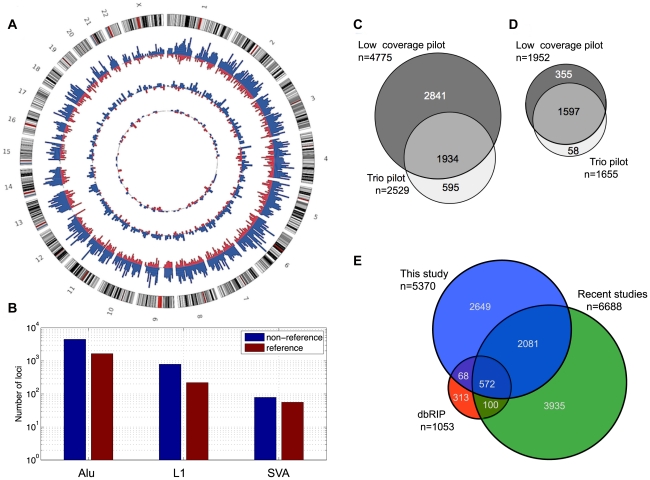
MEI catalog. a) MEI genomic distribution. Circos plot with non-reference MEI represented in blue and reference MEI in red. The outermost ring of chromosomes show the cytoband structure. The outer histogram displays counts of Alu polymorphisms in bins of 5 Mbp, the middle ring L1 polymorphisms in bins of 10 Mbp, and the innermost ring SVA polymorphisms in bins of 20 Mbp. The radial scale of the site counts is the same for each element type. b) MEI family breakdown. Non-reference MEI (blue) and reference MEI (red). c) Venn diagram of non-reference MEI from each pilot dataset. Most of the loci were detected from the low coverage dataset (dark grey). d) Venn diagram of reference MEI from each pilot dataset. e) Venn diagram of non-reference MEI from this study and other studies [23]–[29], [34], [35].

The complete set of 7,310 MEI calls is simply the combined set of reference and non-reference MEI over both pilot datasets (summarized in Table 1, complete list in Table S1). Insertions occurring at the same locus from different call sets were merged using a 100 bp window for matching positions, choosing the SR insertion coordinate when available to represent the merged event. Similarly for reference MEI, deletion merging was accomplished among the 23 separate 1000GP call sets using a precision-aware algorithm described in detail in the 1000GP SV companion paper [30]. The full catalog of MEI loci appear to be distributed randomly across the genome (Figure 2b) with a characteristic spacing of 0.4 Mb between MEI loci, except for an apparent MEI hotspot in the HLA region of chromosome 6 where 19 MEI loci are clustered in a 1 Mb region (8 times the genomic average density for MEI, Figure S11). Accurate read mapping in the HLA region is complicated by a high density of variation [36], however, we see no evidence of falsely detected MEI here. The balance between reference and non-reference MEI, proportions of RP and SR detected loci, the fraction of previously identified MEI loci, and the validation rate are all consistent with genomic averages; only the density of MEI is significantly increased.

The genomic proportions of the three mobile element families are 85±2% Alu, 12±2% L1, and 2.5±1% SVA (Figure 2b) for both reference and non-reference MEI. Most non-reference MEI loci were detected from the low coverage pilot data (Figure 2c) while the reference MEI were more evenly distributed between the low coverage and trio pilot data (Figure 2d). As described in the 1000GP main pilot paper [36], more than 80% of the non-reference MEI were newly identified loci not detected by previous studies [23]–[28], [34], [35], [45]. However, in the mean time, several published studies have produced new lists of non-reference MEI loci including L1 insertions [34] and Alu insertions [28], [35]. Half of the non-reference MEI loci from this study have not yet been reported elsewhere (Figure 2e, Figure S8). Table 1 of the 1000GP paper lists 5,371 MEI, two of these events were subsequently merged into one to form the present count of 5,370 MEI detected as insertions. For reference MEI, we find that 76% of our events matched deletion coordinates listed in the dbVAR (28 January 2011) structural variation database or a deletion identified in the HuRef genome [22], [46], leaving 24% of the reference MEI unreported prior to 1000GP publications.

The 1000GP catalog of MEI variant sites includes all 7,310 detected loci, including those matching MEI from other publications. Further comparisons among the recent MEI studies are provided in Text S2.

### Detection specificity and sensitivity

We benchmarked each of the four non-reference MEI call sets (separate SR and RP call sets for the low coverage and trio pilot datasets) to assess detection sensitivity and specificity. As MEI are currently not suitable for microarray validation due to their highly repetitive sequence, all validations were done by locus-specific PCR. 200 loci were randomly selected from each of the four insertion call sets. Using an automated pipeline [32], primer design was possible for 746 loci (Table S4). In addition to the randomly selected loci, other candidate loci were selected for validation experiments in order to confirm SVA insertions (n = 7), to test potential *de novo* insertions from the pilot 2 trio (n = 1), and gene-interrupting events (n = 86 attempted), as well as for algorithm training and testing purposes (n = 386). These additional PCR results (Table S4a) were not used to assess false detection rates, except for the special case of SVA insertions, which were under-represented in the random loci selection since SVA insertions are relatively rare.

**Figure 3:**
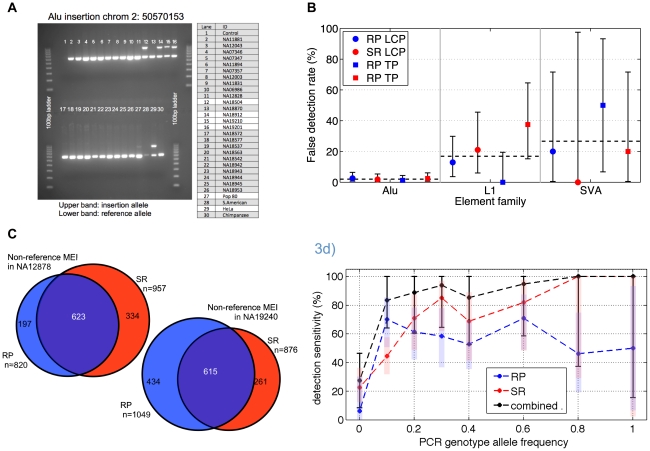
Non-reference MEI validation and detection sensitivity. a) Example of PCR gel chromatograph validation results. At this site, three of the 25 low coverage samples show two bands characteristic of heterozygous insertions. Two additional test samples (Pop80 and HeLa) also show the insertion allele. b) False detection rate estimates based on PCR experiments at random sites, broken down by element type (Alu, L1, SVA), algorithm (RP & SR), and dataset (LCP: low coverage pilot, TP: trio pilot). The false detection rate for Alu elements is uniformly <3% while the false detection rates for L1s and SVA element insertions approach 30%, with large error bars (95% confidence intervals) arising from relatively low statistics. c) Non-reference MEI detection overlap from trio samples NA12878 and NA19240. This level of overlap between two independent methods using independent sequence data corresponds to a detection sensitivity of roughly 70% for each algorithm and a combined detection sensitivity of 90% in these samples. d) Non-reference MEI detection sensitivity as a function of allele frequency in the low coverage dataset. PCR results for loci randomly selected from one method were used as a gold standard for the complementary method, and vice versa. PCR also provides an estimate of the allele frequency based on the 25 low coverage samples used for validation experiments. RP (blue) and SR (red) and the combined (black) detection sensitivities rise with frequency. One standard deviation confidence intervals are shown as shaded bars for the RP and SR algorithm, with black error bars for the combined RP+SR detection efficiency.

All candidate loci with successful primer design were tested on two different population genetic panels (Materials and Methods: Validation) one with DNA of 25 individuals from the low coverage pilot, and one with DNA from all samples of the trio pilot dataset. In addition to other human samples from populations not represented by the pilot datasets, DNA of a chimpanzee was also included on the panel to confirm that the identified insertion is indeed human-specific. An example of typical results for a low coverage locus is shown in Figure 3a. Through additional primer design for loci with inconclusive results and PCRs using a primer residing within the 3′ end of a retrotransposon, in particular within SVA elements, more than 98% of the tested candidate loci were successfully genotyped. The validation experiments revealed overall insertion false discovery rates for each dataset of less than 5% (Table 1). Among the different retrotransposon families (L1, SVA, and Alu elements), false discovery rates varied noticeably (Figure 3b), with Alu insertions showing the lowest false-positive rate (2.0 [1.1–3.4] %, followed by L1s (17 [10]–[27] %), and SVAs (27 [8]–[55] %) with 95% confidence intervals. This is not entirely unexpected as polymorphic Alu insertions tend to be low divergence full-length AluY elements, unlike L1 or SVA insertions which tend to be truncated and may be accompanied by adjacent transduced genomic DNA sequences. Although the SR and RP detection methods are very different, the overall detection specificities were remarkably consistent.

Following the validation of non-reference MEI, we assessed detection sensitivity. The primary challenge here was to find suitable gold standard non-reference MEI that should be present in our samples from which to assess sensitivity. We estimated sensitivity in three different ways, as a consistency check. First, we estimated sensitivity by using the high quality non-reference MEI from HuRef [23] as a gold standard and found that 74% of the 650 Alu, L1, or SVA insertions in HuRef matched MEI insertion loci in our catalog (Table S5). This represents a lower limit for insertion detection sensitivity since not all MEI in the HuRef genome are necessarily present in the 1000GP pilot samples. Next we looked at the overlapping insertion detection between the RP and SR methods in the trio children samples (Figure 3c, Figure S6), which were the samples sequenced to the highest depth for both Illumina and 454 data. Based on the detected loci overlap (see Materials and Methods: Detection sensitivity), we estimate 67%±3% and 70%±7% sensitivities respectively for RP and SR insertion detection in the trio children (Table S6), with a combined SR+RP detection sensitivity exceeding 90% in the CEU trio child (see Materials and Methods, Eq. 4) with high coverage data from both 454 and Illumina reads.

A third approach to estimate for the non-reference MEI detection sensitivity is based on the validation PCR genotypes in the low coverage dataset. Since the PCR loci were selected as random subsets for each RP and SR call set independently, the validated sites selected from SR events can be used as a gold standard to assess RP detection sensitivity, and vice-versa. Detection sensitivity as a function of allele frequency (Figure 3d) was estimated for each method from PCR genotypes at those loci randomly selected for validation of the complementary method. PCR genotypes provided the allele frequency estimate on the abscissa. Statistical errors at high allele frequency are large because the limited number of tested MEI loci at higher allele frequencies. Detection sensitivity of the RP method saturates close to 70% at high coverage and the SR method sensitivity exceeds 70% at high coverage (Figure S6). The corresponding trend is apparent in Figure 3d. The combined detection sensitivity approaches 90% for common alleles (Materials and Methods, Eq. 4). However, since relatively few of the low coverage samples were sequenced with 454, a realistic estimate for the detection sensitivity to common MEI insertions is between 70% and 80%. This is consistent with 75% derived from the HuRef gold standard comparison and the sensitivity estimate from the trio pilot overlaps. Equivalent estimates for Alu, L1, and SVA specific sensitivities for common MEI alleles are 75%±10%, 50%±10%, and 50%±20% respectively (Table S9).

Regarding reference MEI detected as deletions, the overall validation rate from PCR and local assembly for the MEI component of deletions was 96%. This does not imply that the remaining 4% were false, only that the released set of deletions contained reference MEI detected by two high specificity algorithms with characteristic false detection rates less than 10%. These algorithms did not require additional validation evidence in the 1000GP release. A rough estimate for the false detection rate for the MEI component of deletions is therefore 0.4%. The number of algorithms supporting a given call is another indicator of call quality. The average number of separate deletion calls (out of a maximum of 23 call sets) supporting events in the MEI subset was 7.8 while the average over all other deletions was 2.3 (Figure S2). The high validation rate and high consensus among detection algorithms indicate that this subset of deletions is relatively free of detection artifact. The practical limitation on the specificity of these events as reference MEI is the subsequent MEI selection criteria. Only a small fraction the 2,010 selected events were ambiguous in terms of matching coordinates to an annotated mobile element with corresponding gap in the chimpanzee genome assembly (e.g. Figure S3, bottom panel). The 1000GP CNV paper identified 2029 reference MEI variants using the BreakSeq algorithm. Overlap between the respective lists is 89%. We estimate 10% as an upper limit on the false discovery rate for reference MEI.

Detection sensitivity for reference MEI was estimated from the fractions of gold standard reference MEI identified by Xing et al. from HuRef [22], [23], [46], and reference MEI identified by Mills et al. [4], [47] from 1000GP samples NA12878 and NA12156 matched to any of our 2,010 reference MEI (Table S5). In each case the fraction of those MEI deletions found in this study exceeded 90%. This level of detection sensitivity is considerably higher than the bulk deletion detection sensitivity reported in the SV companion paper [30], indicating that the RP and SR deletion detection methods developed for the 1000GP were particularly well suited for reference MEI detection.

### MEI properties, assembly, and sub-family classification

**Figure 4:**
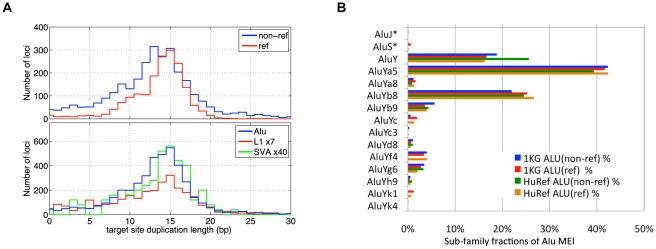
MEI Alu sub-family breakdown, Target site duplication length. a) Length of target site duplications bracketing the MEI sites. Different detection modes (top) and different element families (lower plot) exhibit similar distributions target site duplications lengths. b) Alu sub-family breakdown of 1,105 assembled Alu non-reference insertions. Also shown are the Alu breakdowns from reference MEI (ref) from this study, as well as variant Alus found in the HuRef genome by Xing et al. AluYa5 is the most frequent polymorphic Alu sub-family.

We characterized each detected MEI event (Table S1) by the insertion position, which algorithm(s) detected the event, number of fragments supporting the insertion and reference alleles, insertion length (Figure S12), element family, bracketing homology (Figure 4a), and assembled sequence. MEI have a characteristic “target site duplication” region of homology bracketing the insertion. The target site duplication length distributions for the MEI detected by different methods, as well as for different element families, peaked at 15 bp with a standard deviation of 7 bp (Figure 4a). The full insertion sequence from reference MEI is readily extracted from the reference, but non-reference MEI require local *de novo* assembly to reconstruct the inserted sequence. For this we used 454 data to reconstruct 1,105 Alu insertions (Tables S1 and S7) from our event list based on the PHRAP assembly program [48]. We then used BLAT [49] to map assembled contigs back to the build 36.3 human reference to identify the boundaries of the inserted sequence. The inserted sequence was then mapped back to the RepeatMasker mobile element sequences using the RepeatMasker web server (http://www.repeatmasker.org) to identify the sub-family (Figure 4b). The accuracy of Alu sub-family classification was assessed by comparison to matched 359 Alu insertions from dbRIP [29] and nine fully sequenced Alu insertions from PCR validation experiments. 272 of the assembled Alu sub-family classes were identical (74%). The most active Alu sub-families are AluYa5 and AluYb8. AluY sub-families account for essentially all Alu variation. The relative proportions among Alu sub-families are consistent among reference and non-reference MEI, as well as consistent with the Alu sub-families observed in HuRef [23]. The Alu sub-family breakdown differs from that reported by Hormozdiari et. al. [35] who identified more than 10% of their set of insertions from AluJ or AluS sub-families. The authors of that study point out that these ‘older’ Alu events could arise from mechanisms other than retrotransposon insertions.

### Genotyping

Genotyping of non-reference MEI (Materials and Methods: Genotyping) was based on counts of fragments supporting the reference allele and fragments supporting the insertion allele at each locus for each sample. Heterozygous MEI sites are identified by roughly equal amounts of reference and alternate allele supporting fragments spanning an insertion locus, while homozygous sites should have all fragments supporting one or the other allele. For reference MEI, we used genotypes produced by the Genome STRiP package [39], which was developed for 1000GP deletion genotyping [30], [39] and incorporates Beagle [50] imputation based on linkage with local SNPs. Both genotyping methods provide phred-scaled [51] genotype quality (GQ) metrics at each site that reflect confidence in the given call based on supporting evidence, GQ = 0 to a total lack of genotype evidence and GQ = 10 indicating that the genotype should be 90% accurate. The GQ metric depends on the number of fragments found to support the MEI and non-MEI alleles for a given locus and sample (Text S2: Genotyping methods). As in most issues of sensitivity vs. specificity, there is a trade-off between high genotype efficiency and genotyping accuracy. The drop-off in genotyping efficiency vs. GQ threshold is more severe for non-reference MEI (Figure S13). For subsequent genotype-based analysis of non-reference MEI sites and samples we required GQ≥7, which corresponds to roughly 40% genotyping efficiency in the low coverage pilot data. For reference MEI we required GQ≥10, which corresponds to an efficiency of 80%. Genotyping efficiency improves with increased sample read coverage (Figure S13, bottom panel), particularly for non-reference MEI.

Genotyping accuracy for non-reference MEI is assessed by direct comparison to PCR validation genotypes in the same samples, and by testing for Mendelian errors in the trios and violations of Hardy-Weinberg Equilibrium in the low coverage data (Text S2 Genotyping tests, Figures S13 and S14). Validation genotypes are listed in Table S4 (also as the “VG” field of the released MEI insertion genotyped VCF files). Genotype contingency tables for the low coverage data (Table 3) show an 87% agreement between sequenced genotypes and PCR genotypes for sites with GQ≥7. Genotyping accuracy improves with increasing GQ threshold (Figure S13) but never exceeds 90% in the low coverage data. Non-reference MEI genotyping performance for high coverage trio data (Table 3, Table S8) was considerably better than for the low coverage data. However, for population analyses we used only low coverage data in order to minimize the potential for coverage biases. The accuracy of GenomeSTRiP genotypes (for reference MEI events) with GQ≥10 was estimated at 99% in the full 1000GP deletion call set [30], [36], [39].

### Population segregation of MEI

**Figure 5:**
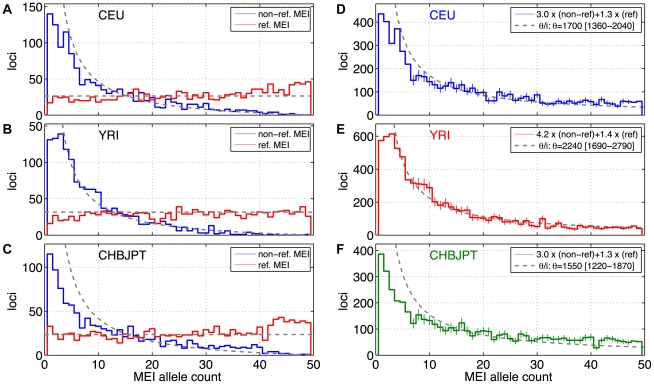
MEI allele count spectrum. a–c) Uncorrected allele count spectra. Non-reference MEI (blue) and reference MEI (red): a) CEU, b) YRI, c) CHBJPT. Loci with 25 or more genotyped samples were included. A random subset of 25 samples was selected for any locus with more than 25 genotyped samples. Gray dashed lines are based on neutral model fits from the full MEI spectra, modified to account for the respective ascertainment conditions, *(θ/2N)* for reference MEI, *(θ/i)(2N−i)/(2N)* for non-reference MEI, where *N* = 25 is the number of samples in the spectrum. d–f) MEI allele count spectra. d) CEU, e) YRI, f) CHBJPT. The spectra are corrected for each detection mode sensitivity and genotyping efficiency according to the expression in the legend. Gray dashed line is a fit to θ/i, where i is the allele count and θ is the diversity parameter. Only counts in the range of 7≤i≤47 were used in the fit (bins with vertical one sigma error bars).

We estimated MEI allele frequencies from the count of high quality (GQ≥7 non-reference and GQ≥10 for reference MEI) genotyped insertion alleles for each MEI locus. Allele frequencies were estimated from loci with at least 25 high quality genotypes for each continental population group. The two MEI detection modes (i.e. reference and non-reference insertions) have very different allele frequency spectra (Figure 5a–5c). Since the non-reference MEI and reference MEI components have very different powers of detection and genotyping, the two components were corrected separately (Materials and Methods: Allele frequency spectra) before being combined into the full MEI spectrum (Figure 5d–5f). We estimated correction factors for each population group, each element type, and each detection mode (Table S9). Non-reference MEI correction factors are larger than reference MEI factors because of the lower detection sensitivity and genotyping efficiency.

The allele count spectra were compared to the standard neutral model [52]–[54], *θ/i*, where *θ* is an MEI diversity parameter and *i* is the allele count in a fixed number of samples. The value of *θ* is fit from the MEI allele count spectrum for each population group and the fitted model is the gray dotted line appearing in Figure 5d–5f. Only allele count bins in the range 0.15<frequency<0.95 were used in the fit (bins marked with error bars in Figure 5d–5f) to avoid regions of poor detection sensitivity. The corresponding gray dashed lines superimposed on Figures 5a–5c also represent the neutral model expectation, modified to account for the respective ascertainment conditions, *(θ/2N)* for reference MEI, *(θ/i)(2N−i)/(2N)* for non-reference MEI, where *N* = 25 is the number of samples in the spectra. These ascertainment condition expressions are based on the assumption that the reference genome represents a random sample from the given population, which is admittedly simplistic but nevertheless explains much of the difference between the allele spectra of reference and non-reference MEI. A coalescent simulation (Text S2 Coalescent, Figure S17) for MEI variation also shows this behavior using standard population history parameters [55]. Fitted values of the diversity parameter *θ* for each of three population groups and each element family are listed in Table 4, along with rough estimates for the corresponding MEI mutation rates based on the neutral model (*μ = θ/(4·N_e_)*) with an effective population size *N_e_* of 10,000 [56], [57]. Confidence intervals for *μ* and *θ* (Table 4) take into account Poisson noise and uncertainties in the correction factors, but do not reflect the degree to which the model assumptions are valid.

**Figure 6:**
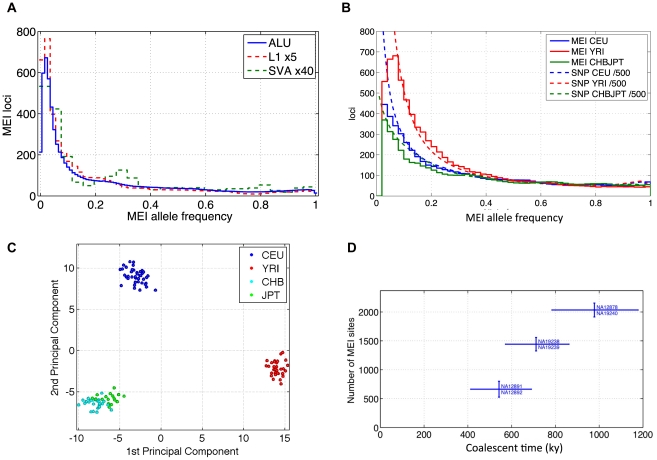
MEI allele frequency spectra, PCA, counts of variants between trio samples. a) Element family breakdown of the combined population allele frequency spectra. L1 and SVA are scaled up to allow comparison with the Alu spectrum. b) MEI and SNP allele frequency spectra across three population groups. The corresponding allele frequency spectra of SNPs relative to the ancestral genome from the 1000 Genomes low coverage pilot project are superimposed as dotted lines. The SNP spectra are scaled down by a factor of 500 for this comparison. c) Principal component analysis of MEI genotypes. CEU: blue; YRI: red; CHB: cyan; JPT: green. The first and second principal components are plotted. d) Total number of MEI between trio samples versus coalescent time based on SNP differences between the sample pairs.

All three element families have been combined into the allele count spectra shown in Figure 5, although the Alu family is the dominant component. Allele frequency spectra for different element families have similar shapes (Figure 6a). We know from SNP studies that the shape of the allele frequency spectrum is modulated by demographic history, and that this shape is characteristically different for European, African, and Asian populations [56], [57]. When compared to SNP allele frequency spectra from the same datasets (Figure 6b), the MEI and SNP frequency spectra show similar trends among the corresponding populations. Among the three population groups, the CHBJPT spectrum shows relatively few low frequency allele loci. This was also apparent in comparison with the neutral model (Figure 5e).

We also analyzed population differentiation by applying principal component analysis to the matrix of allele counts across the low coverage pilot samples and loci (Figures S15 and S16). Some structure is immediately apparent in the matrix of allele counts, e.g. increased heterozygosity in the YRI samples, but PCA reveals population specific patterns of MEI that result in tight clusters of samples according to geographic origin (Figure 6c); again similar to population patterns for SNPs [58], CNVs [59] and deletions [30].

### Functional properties

As few as 39 of the 5,370 non-reference MEI loci were located in exonic sequence, mostly in untranslated regions, and only 3 were found in coding exons (Table 2). These numbers are much lower than expected from random placement (Materials and Methods: Functional calculation), indicating strong selection against MEI disrupting gene function. The suppression factor for an MEI to occur in a coding region compared to the genome-averaged rate is 46x, a much stronger suppression than is observed for coding SNPs (Table S10, suppression factor = 3.9x), and is similar to SNPs that cause the loss of a stop codon (42x, derived from Table 2 of [36]). Two of the MEI interrupting coding regions were PCR-validated. These two MEI appear to be of little functional consequence: ZNF404 is a member of a highly paralogous zinc finger gene family and C14orf166B is a predicted gene without functional annotation. These findings suggest very strong negative selection against MEI interrupting coding regions. Although it is obvious from first principles that insertions in functional regions should be deleterious, the observed suppression factor in a large catalog of MEI in populations quantifies the effect.

**Table 2:** Counts of non-reference MEI contained by annotated function regions.

	Gene	UTR	CDS	Total
**ALU**	1438	32	2	4499
**L1**	249	4	0	792
**SVA**	31	0	1	79
**Total**	1718	36	3	5370
**Expected total**	2020	105	137	-
**Suppression factor**	1.2	2.9	45.7	

Detected events subsequently invalidated by PCR are not counted. Expected counts of insertions were calculated according to random placement across the genome. The p-value that the observed number of CDS interrupting MEI is consistent with random placement is <10^−50^.

**Table 3:** Non-reference MEI genotype contingency tables.

Low coverage pilot					Trio pilot				
		PCR genotypes					PCR genotypes		
Sequenced genotypes		0/0	0/1	1/1	Sequenced genotypes		0/0	0/1	1/1
	0/0	2773	188	5		0/0	901	5	0
	0/1	18	913	217		0/1	2	671	54
	1/1	1	140	372		1/1	0	10	144

Low coverage pilot samples; trio pilot samples. Genotypes are listed in “VCF” convention: 0/0 homozygous reference, 0/1 heterozygous MEI, 1/1 homozygous MEI. For the low coverage validation, 23 samples at 333 sites were tested, while for the trio data all 6 samples were tested at 332 sites. The agreement for the low coverage data is 88.7% with 58% of the sites genotyped with GQ≥7. Genotype agreement for the pilot data was 96% with 90% genotyping efficiency.

### Number of ME polymorphisms between pairs of individuals

The high-coverage trio data allows for the most precise estimates of the total number of MEI variants between pairs of individuals because of the high detection sensitivity. The number of pair-wise variant loci is calculated as the presence or absence of an insertion at a given locus, combining reference and non-reference MEI. We selected the two trio children (NA12878 and NA19240) for comparison between CEU and YRI individuals and the trio parents for comparison of individuals within the CEU and the YRI population groups. After corrections for detection sensitivity and false detection (Text S2 and Table S6), we found 2,034±120 MEI variant loci between the African and the European trio children, 1,442±120 between the YRI parents, and 663±140 MEI between the CEU parents. The pair-wise event numbers scale linearly with coalescent time derived from SNPs (Figure 6d) in these samples (Text S2: Coalescent [60]–[64]).

### Search for *de novo* MEI

Previous estimates for the *de novo* mobile element insertion rate and our own estimate of the MEI mutation rate are one event per 20 births in the human population [23]. Accordingly, we did not expect to find *de novo* insertions in our sample of two trio children. Among all MEI events detected in the trio offspring against the reference (1,778 in NA12878 and 1,971 in NA19240), we did identify a single *de novo* candidate insertion in NA12878, not detected in either parent or in any other sample (Table S6, *de Novo*). A subsequent PCR validation experiment revealed that this insertion was, in fact, present in one of the trio parents, but not detected from the sequence data. All in all, our study found no direct evidence for *de novo* MEI events in the two trio samples.

### MEI heterozygosity and mutation rates

**Figure 7:**
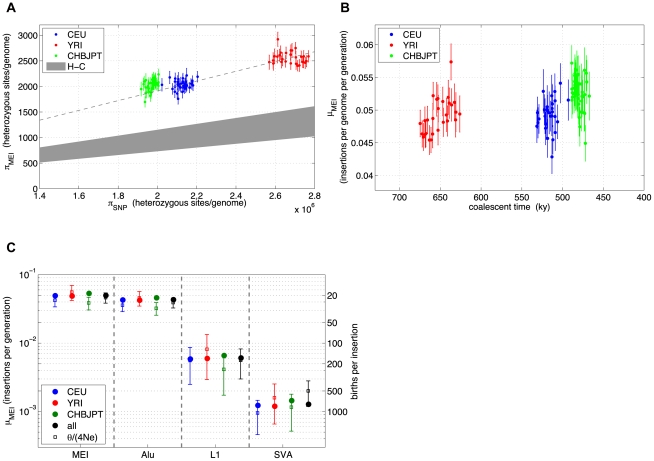
MEI and SNP heterozygosity in low coverage samples. a) MEI *vs.* SNP heterozygosity scatterplot (π_MEI_ *vs.* π_SNP_): The dashed line is a linear model constrained to pass through the centroid of the YRI (red) samples and the origin. The gray region represents an extrapolation from human-chimpanzee (H-C) MEI and SNP differences between the respective genome assemblies. b) Averaged population μ_MEI_ vs. coalescent time scaled to thousands of years, assuming that SNP mutation rate is a steady clock (μ_SNP_∼1.8×10^−8^ mutations per site per generation). c) MEI mutation rates based on heterozygosity (solid circles) and based on allele frequency fits (vertical error bars) for population groups (CEU: blue, YRI: red, CHBJPT: green, all three: black) and estimated separately for element families (all families combined: MEI, Alu, L1, and SVA). Error bars are statistical only.

MEI genotyping allows us to estimate MEI heterozygosity within each sample. We define heterozygosity as the count of heterozygous loci across the individual's genome. In a manner similar to the allele frequency analysis, heterozygosity is corrected for detection and genotyping efficiencies (Materials and Methods: Heterozygosity) such that it represents the true number of heterozygous loci in the sample. Heterozygosity, *π*, and the diversity parameter, *θ*, fit from the allele count spectrum, are related population metrics that depend on the MEI mutation rate, *μ_MEI_*, and demographic history. In the neutral model (under mutation-drift equilibrium in the limit of infinite segregating sites and a constant effective population size, *N_e_*) the two metrics should be approximately equal [65]:
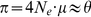
(1)Deviations can be interpreted as evidence for selection pressure, changing demographic parameters, or possibly as changes in the mutation rates. These metrics were originally developed as a framework for SNP analysis but can also be applied to MEI variants. It is this property of heterozygosity that we wish to exploit. A comparison MEI and SNP heterozygosity within the same samples allows a direct comparison of the corresponding mutation rates, because the impact of long-term demography (here simplified in terms of *N_e_*) is identical for both variant types. Consequently, the MEI mutation rate can be estimated as:
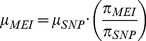
(2)Given constant mutation rates we would expect proportionality between π_SNP_ and π_MEI_ in samples from different population groups, however a scatter plot of π_MEI_ *vs.* π_SNP_ over the low coverage pilot samples (Figure 7a) shows some deviation. Heterozygosity for the Asian sample group is systematically elevated above the proportionality line (dashed line). Also shown on the scatter plot is a grey region corresponding to SNP and MEI differences between the human and chimpanzee reference genomes [37], [66]. The MEI insertion rate is known to be roughly 2.5 times higher in the human than in the chimpanzee lineage [66], however, the time dependence of the MEI mutation rate during human evolution is not yet known. For this, we re-expressed the SNP and MEI heterozygosities for each sample in terms of *μ_MEI_ vs.* coalescent time (Figure 7b) based on equation (2), a constant SNP mutation rate (*μ_SNP_*∼1.8×10^−8^ mutations per site per generation [67]), and the coalescent time estimated from the SNP heterozygosity. Characteristic MEI mutation rates for each population group were derived from Eq. (2) with <*π_MEI_*> and <*π_SNP_*> averaged over the samples in the group. Values of *μ_MEI_* for each population and each element family are compared to *μ_MEI_* derived from *θ* fitting (Figure 7c) and are listed in Table 4 with 95% statistical confidence intervals. Confidence intervals from the allele frequency fits (error bars in Figure 7c) are larger than statistical errors from the averaged heterozygosities over samples (error bars within the circles on Figure 7c) because each sample provided independent observations for the average heterozygosity, whereas in the allele frequency spectra fits all samples were combined. Both estimates are subject to systematic errors that may arise from the detection, genotyping, and correction procedures. To test for systematic biases in *μ_MEI_* we re-processed both allele frequency spectra and heterozygosity estimates over a range of genotype selection thresholds (Text S2: Stability, Figure S18) and found consistent trends in *μ_MEI_* among the population groups and element families, although the overall scales of the mutation rates are uncertain to 20%. Values of the element specific mutation rates in Table 4 and Figure 7c are consistent with previous reports [23], [25], [68]. In summary, careful error analysis led us to believe that the differences in the mutation rate observed between the different population sample groups are likely to result from biological processes, rather than measurement or analytical artifacts.

**Table 4:** MEI population properties.

population	element	Θ [95% CI]	μ(θ) [95% CI]	χ^2^	d. f.	Π [95% CI]	μ(π) [95% CI]
all	MEI	1860 [1540–2170]	0.0464 [0.0384–0.0543]	75.4	78	2160 [2130–2200]	0.0499 [0.0490–0.0507]
CEU	MEI	1700 [1360–2040]	0.0425 [0.0339–0.0510]	52.3	39	2040 [2020–2070]	0.0493 [0.0487–0.0499]
YRI	MEI	2240 [1690–2790]	0.0559 [0.0421–0.0697]	39.9	39	2480 [2430–2530]	0.0488 [0.0478–0.0499]
CHBJPT	MEI	1550 [1220–1870]	0.0387 [0.0306–0.0468]	70.5	39	2030 [2000–2060]	0.0533 [0.0525–0.0541]
all	ALU	1570 [1310–1830]	0.0392 [0.0326–0.0457]	83.9	78	1880 [1840–1910]	0.0432 [0.0424–0.0439]
CEU	ALU	1440 [1150–1720]	0.0359 [0.0289–0.0430]	55.4	39	1770 [1750–1800]	0.0428 [0.0422–0.0434]
YRI	ALU	1830 [1390–2270]	0.0458 [0.0348–0.0569]	43.4	39	2150 [2100–2200]	0.0423 [0.0414–0.0433]
CHBJPT	ALU	1300 [1020–1570]	0.0324 [0.0256–0.0391]	86.5	39	1750 [1720–1780]	0.046 [0.0453–0.0468]
all	L1	224 [120–329]	0.0056 [0.0030–0.0082]	51.9	71	264 [257–270]	0.0061 [0.0059–0.0062]
CEU	L1	223 [100–346]	0.0056 [0.0025–0.0086]	49.6	38	243 [234–252]	0.0059 [0.0057–0.0061]
YRI	L1	326 [118–535]	0.0082 [0.0029–0.0134]	59.6	39	303 [292–314]	0.006 [0.0057–0.0062]
CHBJPT	L1	166 [70–262]	0.0041 [0.0018–0.0066]	49.7	39	251 [243–258]	0.0066 [0.0064–0.0068]
all	SVA	80 [48–113]	0.002 [0.0012–0.0028]	15.4	39	55 [53]–[58]	0.0013 [0.0012–0.0014]
CEU	SVA	38 [18]–[58]	0.001 [0.0004–0.0014]	10.4	27	51 [48]–[54]	0.0012 [0.0011–0.0013]
YRI	SVA	64 [26–101]	0.0016 [0.0006–0.0025]	11.2	24	61 [56]–[65]	0.0012 [0.0011–0.0013]
CHBJPT	SVA	46 [21]–[72]	0.0012 [0.0005–0.0018]	12.5	27	55 [51]–[59]	0.0014 [0.0013–0.0015]

MEI diversity parameter *θ was* fit from the allele frequency spectra for the listed populations and element families. “all” is the full dataset of all three population groups. Insertion rates μ(θ) were derived from the θ values, assuming an effective population size of 10,000. The MEI population heterozygosities π were averaged over samples in the given population group. MEI insertion rates μ(π) were derived from Eq (2) relative to the SNP mutation rate. All insertion rates are listed in units of insertions per genome per generation.

## Discussion

### Common MEI polymorphisms in the human population

MEI alleles propagate within population groups much like other predominantly neutral polymorphisms. MEI allele frequency spectra from the low coverage samples are in general agreement with expectations from the standard neutral model for allele drift in a population. The major differences in allele frequency spectra between non-reference and reference MEI (Figure 5a–5c) are explained by the ascertainment condition that the derived MEI allele occurs in a given sample (the reference) and are in agreement with expectations based on a coalescent simulation of MEI population drift (Figure S17). MEI allele frequency spectra among the three population groups exhibits a similar trend to SNPs (Figure 6b), although the MEI spectrum in the Asian samples is a poor fit to the *θ/i* form (χ^2^/d.f.∼2 from Table 4) with an excess of high frequency alleles and a deficit at low frequency (Figure 5e).

MEI allele frequencies were based on MEI detected and genotyped across three element families (Alu, L1, and SVA), from both non-reference and reference MEI, and multiple detection methods (RP and SR), each with characteristic detection sensitivities and false detection rates. Corrections for these effects, as well as genotyping efficiencies, were included in the allele frequency spectra.

Measurements of MEI heterozygosity offer a more direct method to estimate MEI insertion rates. Like the allele frequency spectrum, heterozygosity is dependent on accurate genotyping and includes corrections for efficiency losses, but in this case the corrections were made on a per sample basis, which is more specific since sample coverage is the dominant limitation for detection and genotyping power (Figure S6). The heterozygosity measurement also has an advantage in that each sample is an independent estimate of the population average <*π_MEI_*> and <*π_SNP_*>. The heterozygosity measurements revealed evidence for differential MEI mutation rates among the three population groups. The probability that the Asian population samples have the same MEI mutation rate as the other two population groups is very low (paired t-test p-value<10^−6^). We tested the stability of this result by varying the genotype selection criteria across a range of threshold (Figure S18) and found that the differential MEI rate effect is indeed stable. Sequence coverage in the 1000GP low-coverage pilot data was roughly the same for all three continental population groups (Table S2), so we do not expect coverage differences to generate significant systematic biases in these population comparisons.

The question remains whether the differential MEI mutation rate between populations is driven by a shared increase of *μ_MEI_* within *Homo sapiens*, as suggested by Figure 7b, or simply by varying insertion rates among different populations. The pilot data is consistent with either interpretation, so data from more populations (more than 30 population groups from five continents are planned for the full 1000GP) will be needed to discriminate between the two hypotheses.

Based on the global values for the diversity parameters *θ_MEI_* and *π_MEI_* (Table 4), and the neutral model, our rough estimate of the total number of MEI segregating sites in the human population with allele frequency>10% is 4500, and 9000 for frequency>1%, with 20% uncertainty arising from parameter estimates. Counting only those sites with a sufficient number of genotypes to measure allele frequency, our dataset contains more than half of the segregating human MEI sites with frequency>10%.

### Significance

This study of the 1000GP pilot datasets is a sizable step toward a complete population-based catalog of common human MEI polymorphisms, made possible by targeting both non-reference and reference MEI events in the human genome. We identified 7,380 polymorphic mobile element insertions from the Alu, L1, and SVA families. Based on experimental validation of random subsets of loci we estimate that the false discovery rate in this study is less than 5%. Detection power for common alleles (allele frequency>10%) varies between non-reference MEI (70%–80%) and reference MEI (>90%). We were also able to assemble the inserted sequence for more than 1,000 non-reference Alu MEI and found consistent proportions of Alu sub-families in comparison to MEI identified in HuRef.

This comprehensive variant discovery and genotyping effort allowed us to directly compare the segregation properties of different variant types from the same dataset. Our analysis revealed that, to a first approximation, the evolution of MEI variants is similar to SNPs and consistent with neutral models [52], [53], except in exonic regions where they are subject to negative selection on the scale that acts against SNP variants resulting in stop codon loss. An intriguing finding from our data, however, is the detection of signals suggesting a recent increase in MEI rates in humans.

## Materials and Methods

### Non-reference MEI detection

Both the SR and the RP methods were based on identification of non-reference MEI as clusters of mapped DNA fragments in which one end mapped to the consensus sequence of a mobile element while the other end was uniquely mapped to the reference genome in a location inconsistent with a known mobile element location in the reference (Figure 1a–1b). The RP method required at least two MEI supporting fragments across both the 5′ and 3′ insertion breakpoints for each candidate MEI from the pooled datasets (the low coverage and trio pilot data were pooled separately). The SR method required only one MEI supporting fragment across either the 5′ or 3′ breakpoints for candidate events. We used 52 consensus element sequences from Repbase [69] (www.girinst.org, version 14.03, Table S11) to identify reads mapping to mobile elements. The RP method used Mosaik [70] (bioinformatics.bc.edu/marthlab/Mosaik, version 0.9.1176) for read pair mapping of Illumina paired-end data to the NCBI36 human reference (build 36.3) and the Spanner [40] program to identify non-reference MEI by clustering supporting fragments [40], [71], [72]. The SR method also used Mosaik to align 454 data, for full read mapping as well as for split-read mapping. We used extensive simulation experiments [73] to optimize detection methods, algorithm parameters, and post-process MEI candidate event selection filters (further details are provided in Text S2).

### Reference MEI selection

The 2,010 reference MEI events are a subset of the 1000GP pilot release of 22,025 deletions [30]. 95% of the MEI sites detected as deletions were found by more than one algorithm but the dominant mapping algorithms were Mosaik, and Maq [74], with detection algorithms Spanner, Pindel [41], BreakDancer [38], and GenomeSTRiP [39]. Two selection criteria ensure that a given deletion corresponds to a true variant MEI inser a given deletion corresponds to a true variant MEI inserted in the reference genome:

1. The deletion coordinates match to an annotated Alu, L1, or SVA element [6] in the hg18 reference, defined as >50% reciprocal overlap and the start and end coordinates both match within a window of 20 bp for Alus, or 200 bp for L1s and SVAs.
2. At least 75% of the deleted region corresponds to a gap in the chimpanzee genome assembly [37].

### MEI event matching between algorithms and studies

Non-reference MEI detected by the SR and RP methods were merged according to a 100 bp matching window around the leftmost insertion coordinates. To assess call set intersections between this study and other published lists of non-reference MEI, we used a matching window of 200 bp around each insertion position. We adopted the ‘leftmost’ coordinate convention (Figure S1), in keeping with 1000GP call sets, whereas other studies used rightmost or unclear coordinate conventions. The respective scales of the matching windows were dictated by the characteristic position resolutions of the algorithms (Figure S7, Figure S10), which varied considerably from study to study. Redundant loci from recent publications were not counted multiple times in Figure 2e. To identify matching reference MEI to other studies we required at least 50% reciprocal overlap between the starting and ending NCBI36 deletion coordinates.

### Calculations of sample sequence coverage

For SR detection the relevant coverage statistic is 454 base coverage, counts of aligned reads covering a given base, averaged across the accessible genome. For RP detection the driving coverage statistic is Illumina read-pair spanning coverage, counts of fragments in which the non-sequenced segment of the fragment between the reads cover a given base, averaged across the genome (Table S2).

### Validation methods

The four non-reference MEI event lists (Table 1) were submitted to the 1000 Genomes Structural Variation subgroup for validation experiments to assess false detection rates. 200 loci from each list were randomly selected for primer design and subsequent PCR validation. Primers were designed as described previously [32], [36] to span across the insertion breakpoint and to guarantee unique mapping to build 36.3. In addition to the estimation of the false detection rates, validation genotypes were derived from gel-band size comparison for each sample and site tested by PCR. We also used the validation data to estimate detection sensitivity based on the overlap of events called between the two independent sequence data platforms and algorithms.

For loci with ambiguous PCR results, no amplification, or amplification of only the empty insertions site, a second primer pair was designed. For the primer design, 600 bp of flanking sequence on either side of the insertion site was retrieved from genome.ucsc.edu using Galaxy. Alu elements within the flanking sequence were masked to “N” using RepeatMasker (repeatmasker.org). Primers were designed with BatchPrimer3 v2.0 in the flanking sequence, leaving at least 100 bp before and after the predicted insertion site. Next, all primers were tested with BLAT to determine the number of matches in the human genome. If one primer of a primer pair matched several times and the other primer was unique, a virtual PCR was performed. Primer combinations with one predicted PCR product were tested on our panel. Otherwise primers were designed manually (if possible) after repeat-masking the flanking sequence with the complete repeat library.

In addition, for L1 and SVA loci without unambiguous PCR amplification, primers were designed, placing one primer within the 3′ end of the mobile element sequence [75]. The primers were designed to match the consensus sequences of the youngest L1 and SVA sub-families. All PCR primers were ordered from Sigma Aldrich, Inc. (St. Louis, MO). All LSU-designed PCR primer sequences used in this project can be found at http://batzerlab.lsu.edu.

#### DNA samples for PCR verification

A subset of 25 DNA samples from the low coverage pilot samples and all six trio samples were used in PCR validations (Table S4). Each DNA panel also included a population out-group sample, an individual of South American origin (NA17310, Coriell) for low coverage pilot, and an individual of Asian origin (NA17081, Coriell) for the trio pilot. Additional control DNA samples on both panels included human cell line DNA, (HeLa; ATCC CCL-2) as well as “Pop80”, a locally pooled DNA sample from different individuals of diverse geographic origins (Asia, Africa, South American, and European). This sample serves as a diagnostic tool because amplification of an empty site alone in all samples (including Pop80) strongly indicates that the putative insertion is absent (false positive). In contrast, the presence of an MEI in a single study subject, while absent in Pop80, points toward a potential *de novo* retrotransposon insertion, or at least an insertion with a low allele frequency (AF). Chimpanzee DNA (NS06006, Coriell) was also included on each panel, representing the presumptive pre-insertion site for each event (empty site) as another PCR control.

In addition to the subset of 25 individuals used for the low coverage pilot validations, four more DNA samples from the low coverage pilot dataset were obtained for subsequent experiments. DNA samples NA12872, NA12814, NA12815 and NA12044 (CEPH/Utah USA) were purchased from the Coriell Institute for Medical Research. All 35 samples (25+6+4) were used for PCR validations associated with MEI events detected specifically in exons.

#### PCR details (LSU)

PCR amplifications were performed in 25 µl reactions in a 96-well format using either a Perkin Elmer GeneAmp 9700 or a BioRad i-cycler thermo-cycler. Each reaction contained 15–50 ng of template DNA; 200 nM of each oligonucleotide primer; 1.5 mM MgCl_2_, 1× PCR buffer (50 mM KCl; 10 mM TrisHCl, pH 8.3); 0.2 mM dNTPs; and 1–2 U *Taq* DNA polymerase.

Full-length L1 and SVA elements typically exceed the limitations of standard DNA *Taq* polymerase in PCR. For L1 insertions, LA-*Taq* DNA polymerase (Takara Bio USA, Clontech Laboratories, Inc. Mountain View, CA) was used in the PCR reactions according to the manufacturer's instructions to enhance the yield of long PCR templates (2–10 kb). SVA elements are particularly GC-rich and difficult to amplify in PCR if full-length even with special long-template polymerases. In order to evaluate presence/absence of these insertions using PCR, we performed a PCR using one primer residing within the SVA insertion in conjunction with an external primer (forward or reverse, depending on the orientation of the predicted insertion). To determine the genotype and presence of the insertion, two separate PCR reactions were required in these instances. A PCR using primers flanking the MEI amplified a PCR product if the MEI was “absent.” A separate PCR with internal primers detected the MEI “present” site. In addition, this approach was also used for some L1 loci to confirm the presence/absence of the insertion and to minimize the chance of false non-detection.

PCR experiments were carried out in three different laboratories yielding similar success rates. At EMBL, PCRs were preformed using 10 ng of NA12878 genomic DNA (Coriell) in 20 µl volumes in a C1000 thermocycler (BioRad). Two different enzymes, iProof High Fidelity DNA Polymerase (Biorad) and Hotstart Taq (Qiagen) were used, with comparable results. PCR conditions for iProof were: 98°C for 1 min, followed by 5 cycles of 98°C for 10 s, 68°C for 20 s and 72°C for 4 min and 30 cycles of 98°C for 10 s, 66°C for 20 s and 72°C for 4.5 min, followed by a final cycle of 72°C for 5 min. PCR conditions for HotStart Taq were: 94°C for 15 min, followed by 5 cycles of 94°C for 30 s, 60°C for 30 s and 72°C for 3 min and 30 cycles of 94°C for 30 s, 56°C for 30 s and 72°C for 3.5 min, followed by a final cycle of 72°C for 5 min. PCR products were analyzed on a 1% agarose gel stained with Sybr Safe Dye (Invitrogen) and a 100 bp ladder and 1 kb ladder (NEB).

PCR reactions at Louisiana State University were performed under the following conditions: initial denaturation at 94°C for 90 sec, followed by 32 cycles of denaturation at 94°C for 20 sec, annealing at 61°C for primers designed by pipeline or 57°C for other primer design for 20 sec, and extension at 72°C for 30 to 90 sec depending on the predicted PCR amplicon size. PCRs were terminated with a final extension at 72°C for 3 min. When LA-*Taq* DNA polymerase was used to amplify L1 insertions, the extension step of each cycle was carried out at 68° for 8 min, 30 sec, followed by a final extension step at 68° for 10 minutes at the end of the run. 20 µl of each PCR product were size-fractionated in a horizontal gel chamber on a 2% (*Alu* and SVA) or 1% (L1) agarose gel containing 0.1 µg/ml ethidium bromide for 60 minutes at 175 V or 1 hour/45 min at 150 V, respectively. UV-fluorescence was used to visualize the DNA fragments and images were saved using a BioRad ChemiDoc XRS imaging system (Hercules, CA).

An outcome from the validation experiments on the 86 gene-interupting MEI was a high false detection rate for candidate Alu insertions in close proximity to 7SLRNA annotations. Subsequently we reclassified all 22 Alu insertion candidates within 200 bp of a 7SLRNA as invalidated (Table S1).

### Detection sensitivity

The two non-reference MEI detection methods use independent DNA libraries. So the overlap between the RP and SR are governed by the respective detection sensitivities, statistically akin to the Lincoln-Peterson method [76] used in ecological studies to estimate the size of a population based on two random capture and recapture samplings. This estimate assumes that the two algorithms are sensitive to the same type of events and that the difference between the event lists is a sampling issue. The expression for the detection respective detection sensitivities (*ε_RP_* and *ε_SR_*) depends on the false detection rates (*f_RP_* and *f_SR_*) provided by the validation experiments, the counts of loci detected by each method (*n_RP_* and *n_SR_*), and the count of loci detected by both methods (*n_RP_._SR_*):
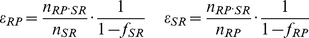
(3)Given detection sensitivities *ε_RP_* and *ε_SR_* from independent datasets and methods, the combined detection sensitivity (*RP+SR*) becomes:
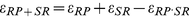
(4)for samples in which both types of data were available (e.g. trio samples NA12878 and NA19240).

### Genotyping methods

For reference MEI we used available genotypes calculated by GenomeSTRiP [39] for the 1000GP deletion call set. GenomeSTRiP results were not readily available for non-reference MEI so we developed a simple Bayesian framework to estimate the posterior probability for each possible genotype. The posterior genotype probability is:
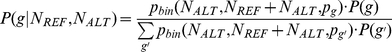
(5)where *N_ALT_* and *N_REF_* are the counts of fragments supporting the alternate and reference alleles respectively; *g* is the genotype (i.e. homozygous reference allele, heterozygous, homozygous insertion allele); *P(g)* is the prior probability for the genotype *g* (a flat prior was used, *P(g) = 1/3*); *p_g_* is the expected fraction of insertion fragments given a genotype g (i.e. *p_g_ = 0.5* for heterozygous insertions, *p_g_∼0* for homozygous reference, and *p_g_∼1* for homozygous insertions); *P_bin_(N_ALT_,N_ALT_+N_REF_,p_g_)* is the binomial probability that *N_ALT_+N_REF_* fragments will fluctuate to *N_ALT_*, given an expected fraction *p_g_*. The called genotype for a given site is the genotype with the maximum posterior probability. The Bayesian framework also provides genotype likelihoods, which are used to construct genotyping quality metric (*GQ*) for each site and sample. The *GQ* value adopts the “phred” quality convention:
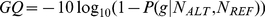
(6)Where *P(g|N_ALT_,N_REF_)* is the posterior probability for the called genotype from Eq. (5). GQ is highly dependent on the total number of supporting fragments (reference plus insertion). A selection of sites at *GQ* = 7 should correspond to roughly to 80% genotyping accuracy and corresponds to sites with 2 or more supporting fragments.

### Allele frequency spectra

MEI loci with at least 25 genotyped samples per population (50 samples for the combined population spectra) were included in allele frequency spectra. Sites of GQ≥7 non-reference MEI and of GQ≥10 reference MEI were included. For loci with more than 25 genotyped samples, a random subset of 25 was used for the allele count spectra (Figure 5). For the allele frequency spectra (Figure 6a–6b) we projected down to 25 samples according to the hypergeometric distribution [56], [57] which smooths the spectrum while retaining all available information from loci with more than 25 genotyped samples. Hypergeometric projection was not used to build the allele count spectra used for fitting purposes because it introduces correlation among allele count bins. We constructed the allele count spectra for MEI events detected as insertions and those detected as deletions separately to account for the distinct ascertainment conditions before combining them into the aggregate spectrum. The combined spectrum includes corrections for respective detection and genotyping efficiencies:
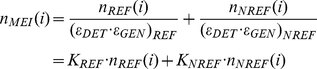
(7)where *n_REF_(i)* and *n_NREF_(i)* are the counts of genotyped loci for reference (e.g. Figure 5b) and non-reference MEI (Figure 5a) at allele count *i*, *K_REF_* and *K_NREF_* are scaling factors for each detection mode (not dependent on *i*), and *n_MEI_(i)* is the net count of MEI variant loci at a given allele count *i* (Figure 5c–d). The correction factors depend on the detection sensitivity (ε_DET_) and genotyping efficiency (ε_GEN_) as K = (ε_DET_·ε_GEN_)^−1^. Genotyping efficiency is simply the fraction of detected sites with 25 more genotyped samples (Table S9). Detection efficiency is described above (Detection specificity and sensitivity, Figure 3d). SNP allele frequency spectra (Figure 6b) were based on the 1000GP release VCF files (ftp://ftp-trace.ncbi.nih.gov/1000genomes/ftp/pilot_data/release/2010_07/) with no corrections. SNP allele frequency spectra were projected down to 50 samples using the hypergeometric distribution [56], [57].

### Functional calculation of suppression factor

Only non-reference MEI with insertion position confidence intervals entirely within annotated regions (Gene, UTR, CDS) were counted. No MEI that were subsequently invalidated were counted. Relative to random placement across the genome the MEI suppression or boost factor is defined as:
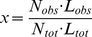
(8)where *N_tot_* is the total number of MEI loci, *L_tot_* = 2.85×10^9^ bp is the length of the accessible genome, *L_obs_* is the size of the region (1 MB or the sum of coding regions) where the number of observed MEI is *N_obs_*. The null model for MEI placement results in a binomially distributed *N_obs_*, which is generally not far from what we observe, except in the case of functional regions (suppressed) and HLA (hotspot). For the calculation of MEI inserted in CDS regions, only non-reference MEI were considered, since an embedded reference MEI precludes annotation as a coding sequence.

### Heterozygosity

MEI and SNP heterozygosity for each sample were calculated from the counts of genotyped heterozygous sites. For MEI, the total numbers of genomic heterozygous sites were estimated with corrections for genotyping efficiency and detection sensitivity. The genotyping efficiency for a given sample is the fraction of detected loci with high quality (GQ≥7 non-reference, of GQ≥10 reference MEI) genotypes. There is also a sample specific correction for genotyping bias against heterozygotes at sites with limited fragment coverage:
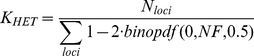
(9)where the sum is over genotyped loci passing the GQ threshold for the given sample, *N_loci_* is the count of such sites, *NF* is the count of supporting fragments (both reference and insertion allele) at the site and *binopdf* is the binomial probability density function that a heterozgygous site will randomly produce only reference supporting fragments. The *K_HET_* correction was applied only to the non-reference MEI component because, for reference MEI detected as deletions, GenomeSTRiP used not just supporting fragment information for genotype likelihoods, but also used Beagle to impute missing data from linkage with local SNP haplotypes to identify heterozygous deletions. For each sample (*s*) the number of heterozygous MEI in the genome is estimated as:
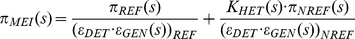
(10)where *π_MEI_(s)* is the heterozygosity for sample s, *π_REF_(s)* and *π_NREF_(s)* are the raw counts of heterozygous sites for reference and non-reference MEI, ε*_DET_* is the detection sensitivity, and ε*_GEN_(s)* is the fraction of detected sites genotyped in the given sample (Figure S13, Table S9). SNP heterozygosity is derived from the raw counts of heterozygous sites. All values of heterozygosity are in units of heterozygous sites per genome, and the length of the genome is considered to be the accessible genome (2.85 Gb) [36].

The SNP heterozygosity values are transformed to rough estimates of the corresponding coalescent time (Figure 7b) [77]:
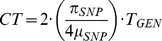
(11)where, μ_SNP_ = 1.8×10^−8^ mutations per site per generation, and *T_GEN_∼25 y* is the average time between generations.

## Supporting Information

Figure S1 Insertion coordinate convention.
(EPS)

Figure S2 Number of deletion call sets supporting reference MEI locus. The average number of deletions call sets supporting MEI events is about eight (blue) while for all deletions in the 1000GP release (gray dashed line) the average number of calls was about three. The peak at the call sets for Alu MEI deletions corresponds to the eight Illumina RP based call sets (BC, Wash U, WTSI, for both pilots, Broad for pilot 1 and U.Wash for pilot 2) and two SR call sets (Pindel for both pilots).
(EPS)

Figure S3 UCSC browser display of reference MEI. (top) The deletion (red track with 1000GP deletion id's P1_M_061510_12_213 for low coverage pilot and P2_M_061510_12_22 from the trio pilot) matches to the annotated AluYg6 element at chr12:8516855–8517156, present in the NCBI36 reference sequence but missing in the sequenced sample. The black RepeatMasker track shows that the AluYg6 element matches the deletion start and end coordinates. The green tracks indicate the extent of the chimpanzee assembly, which does not include the AluYg6 element. The blue DGV tracks show that this particular deletion has been previously identified by several experiments with various degrees of position resolution. (bottom) Example of questionable reference MEI. The blue track at the top marks a detected deletion (id P2_M_061510_3_301) at chromosome 3, 60,660,331 bp that overlaps >50% with a short annotated L1HS element, but the start and end coordinates do not match precisely. The chimpanzee genome (in yellow) has a gap in the region, but the edges do not align precisely. This deletion was included in the count of 2,010 reference MEI, but adds to the level of uncertainty.
(TIFF)

Figure S4 1000 Genome Project pilot sample breakdown. a) Venn diagram of pilot samples by sequencing platform (Illumina and 454 only). The bulk of the samples were sequenced by Illumina. The circle areas are only roughly proportional to the number of samples contained. b) Venn diagram of samples used for MEI detection (left) and genotyping (right). MEI detected as insertions (red) and deletions (blue) have different signatures and algorithms resulting in the difference between the samples used.
(TIFF)

Figure S5 Illumina paired end fragment length distributions. Left) Low coverage pilot fragment length distributions for a random selection of 20 lanes of Illumina read pair data. Most libraries have a median fragment length from 100 to 300 bp with a wide variety of shapes. Right) Trio pilot fragment length distributions for 130 lanes of Illumina read pair data for NA12878. Five libraries are shown in different colors with different characteristic shapes. The small peak visible in orange at 550 bp is shifted by 300 bp from the main peak. This small peak arises from reference Alu insertions of length 300 bp. This small Alu peak occurs for all libraries in both pilots.
(EPS)

Figure S6 MEI insertion sensitivity vs. coverage for the two methods. Coverage for the RP method is quantified as “span” coverage on the blue scale. Span coverage is calculated based on the fragment gap between the reads at the end of the fragment where RP detection is sensitive to large structural variations. The SR algorithm sensitivity depends on read coverage (red scale at the top) because the insertion can be detected anywhere within a given read (except within 20 bp of the ends). The detection sensitivity at maximum coverage is determined by the trio overlap calculations from Table S6. Sensitivity at reduced coverage values is calculated by down sampling the number of supporting reads and counting the fraction of insertions that survive the selection criteria.
(EPS)

Figure S7 Non-reference MEI insertion breakpoint resolution. (top) the position residual between matched RP to SR insertions. (bottom) 1000GP loci vs. dbRIP. The dbRIP hg18 coordinates were shifted by TSD such that both lists adopt the ‘leftmost’ coordinate convention.
(EPS)

Figure S8 Venn diagrams of MEI insertion overlap with recent studies. (top) L1 overlap with Ewing and Kazazian [34]. (bottom) Alu overlap with Hormozdiari et. al. [35].
(EPS)

Figure S9 Genomic distance to nearest element of the same family. (top) Non-reference MEI. 1000GP and HuRef distributions are plotted as well as L1 distances for Ewing and Kazazian [34] and Alu distance for Hormozdiari et. al. [35]. Distances <1 indicate insertions within annotated elements.
(EPS)

Figure S10 Insertion position resolution comparison. Non-reference MEI were matched to dbRIP using a 200 bp window.
(EPS)

Figure S11 Number of MEI per 1 MB binned regions across genome. (top) Dotted gray line is a simple Poisson model for MEI distributed uniformly across the accessible genome (2.85 Gb). The red arrow points to a significant hotspot in chromosome 6, position 33 Mb in the HLA region where 19 MEI were detected in a 1 MB region. (bottom) MEI density profile across chromosome 6 showing spike in region of HLA at 33 Mb.
(EPS)

Figure S12 MEI insertion length. a) Comparison of insertion lengths with 617 dbRIP assembled MEI insertions that match 1000 Genomes MEI using a 200 bp window around insertion position. b) MEI insertion length residual distribution. c) The insertion length from MEI deletions (red) is the number of reference nucleotides in the deleted region (the annotated mobile element plus one copy of the TSD and any carry-over sequence). Sharp peaks at 300 bp and 6000 bp are the Alu and L1 insertions respectively. The insertion length for MEI detected as insertions (blue) is estimated from the span of the mapping coordinates within the mobile element. This estimate does not take into account any inserted sequence that is not part of the mobile element such as the TSD, poly-A tail, or carry-over sequence.
(EPS)

Figure S13 Genotyping efficiency. top) Fraction of MEI sites surviving genotype quality thresholds in low coverage data for non-reference MEI (blue steps, GQ≥7) and for reference MEI (red, GQ≥10). Also shown is genotype accuracy based on validation experiments for non-reference MEI (dashed with grey 95% confidence interval). bottom) Sample-by-sample fraction of MEI sites surviving genotype quality threshold for vs. coverage in low coverage samples. Non-reference MEI (crosses) show a genotyping efficiency approaching 60% at 4 fragments/base spanning coverage, while reference MEI (circles) genotyping efficiency is nearly flat at 80%. Samples from the three population groups show the same trends. Coverage here is calculated as spanning coverage, most relevant for RP detection.
(EPS)

Figure S14 Hardy-Weinberg Equilibrium test. Proportions of each genotype as a function of allele frequency for each population group (blue: CEU, red YRI, and green CHBJPT). Also plotted in gray dashed lines for comparison is the proportion expected from HWE.
(EPS)

Figure S15 Genotype Matrix of low coverage samples. Each element in the matrix corresponds to a sample and a locus at which the genotype is color coded. Sample populations are labeled across the top, separated by green lines. The chromosome order for the MEI loci is labeled on the right side, with non-reference MEI (“insertions”) and reference MEI (“deletions”) grouped separately. This matrix was input to Principal Component Analysis for plotted in the main text Figure 6c (Figure S16d).
(EPS)

Figure S16 Principal Component Analysis population clustering for PCR genotypes, MEI ins, MEI del, combined. A matrix of genotypes for each site and sample was input to a PCA and the resulting first two components are plotted against each other. The sum of insertion alleles is the value in the matrix elements. For elements corresponding to sites and samples without genotypes, the global average genotype value was used. a) Genotypes from PCR validation for the low coverage pilot. b) Genotypes from low coverage non-reference MEI only. c) Genotypes from reference MEI only. d) Genotypes from samples with both non-reference and reference MEI. Population clusters become tighter as more MEI insertion information is added to PCA.
(EPS)

Figure S17 Coalescent simulation allele frequency spectra for the combined CEU, YRI, CHB and JPT population groups. AF is binned in units of 0.1. The lowest bin (0–0.1) is not plotted to allow the spectra at higher AF to be compared. The normalizations for MEI detected as insertions (red) and deletions (green) are set to that the two components sum to the total unbiased MEI AFS (blue).
(EPS)

Figure S18 MEI insertion rate vs. coalescent time for increasing MEI site selection thresholds. The estimated MEI insertion rates (main text Eq.2) for each sample is plotted vs. the coalescent time derived from SNP heterozygosity. Panel a) is the same as Fig. 7b from the main text and corresponds to genotyped sites with GQ≥7, which also corresponds to sites with at least two supporting fragments. As more supporting fragments are required b) NF≥3, c) NF≥5, d) NF≥7, the numbers of genotyped sites decrease, but the trend between populations in the MEI insertion rates remains.
(EPS)

Table S1 Combined MEI event list (external Excel file). Genomic coordinates with confidence intervals are listed for each of the 7380 MEI loci. Each event is characterized by an element type (ELEMENT = Alu, L1, or SVA), element STRAND (+ or −), detection (DET = DEL or INS for non-reference and reference MEI respectively), event ID, estimated insertion length (LEN), detection algorithm (ALG), validation status (VAL), validation method (VALMETH = PCR, ASM for assembly, 7SLRNA should be discarded due to proximity to annotated 7SLRNA element), population (POP = CEU, YRI, CHB, or JPT), allele frequency in three major groups (AF), number of genotyped samples in the three groups, number of insertion alleles in the three groups, previous study ID's (DBVARID, DBRIPID, PUBID), TSD length, number of insertion-supporting fragments from the 5′ side (NALT5), from the 3′ side (NALT3), the 1000 Genomes CALL SET name, quality value (Q), gene/exon/UTR/CDS interrupted (GENE), sub-family, and inserted sequence when available, and a list of all samples in which the alternate allele was detected (ALTSAMPLES). Note: 71 events identified by the VAL field as invalidated or in close proximity to a 7SLRNA loci are marked in yellow and were not included in the counts of interrupted genes, exons, UTRs, or CDS regions.
(XLSX)

Table S2 Samples with corresponding sequence coverage (external Excel file) Sequence coverage for each of the 185 samples calculated in terms of Illumina span-coverage for RP detection, 454 base coverage for SR detection and Illumina base-coverage (including single-end read data) for deletion detection.
(XLSX)

Table S3 Reference MEI detection method breakdown. (external Excel file) Thirteen different algorithms contributed to the detection of MEI present in the reference but not in a sample. a) Breakdown by pilot. b) Breakdown by algorithm. The bulk of MEI deletions were found by Illumina RP and SR methods.
(XLSX)

Table S4 Validation genotypes for non-reference MEI datasets (external Excel file). Complete genotyping information for all samples tested at the 746 sites used for false detection rate estimates and for genotyping assessment. a) Additional validation results for non-reference MEI loci (external Excel file) Genome coordinates for 267 additional validation PCR experiments carried out at Yale, EMBL, and LSU. These experiments were done as preliminary tests (EMBL, Yale, LSU-PRELIM) and for testing specific loci (SVA, *de novo*, exon interrupting).
(XLSX)

Table S5 MEI sensitivity based on comparison to gold standard events. (external Excel file) The fraction of HuRef MEI [23] found by this study is a lower limit to the detection sensitivity to common MEI alleles. a) MEI insertion detection sensitivity. b) MEI deletion sensitivity. b) MEI deletion sensitivity based on loci detected in the same samples from Mills et al. [47].
(XLSX)

Table S6 Trios (external Excel file). a) Overlap between RP and SR in the same trio samples (NA12878 and NA19240) can be used to estimate detection sensitivity. Columns RP and SR are the counts of all loci for the two samples broken down by element type. RP-only and SR-only count loci where only one method found the insertion. RP+SR is the count of loci deleted by both methods. The detection sensitivity estimates (ε_RP_, ε_SR_, and ε) with corresponding statistical 1-sigma errors are derived from the overlaps. The combined detected efficiency is based on the union of the two independent methods. b) Counts of MEI site differences between two individuals. The trio samples were used for this because of the relatively high coverage and corresponding sensitivity to low frequency alleles. Corrections to the counts compensate for less-than-perfect detection sensitivity and false detections. The trio children from two populations (CEU and YRI) have the most differences (2034±120) while the CEU parents have the fewest (663±120). The YRI parents' count of sites is between the other pairs. These differences are plotted vs. the corresponding coalescent time in Figure 6d (main text). c) *De novo* insertion hunt. Any MEI appearing in the children of the family trios but not in the parent would be a *de novo* MEI insertion. Six candidates from NA12878 (a) and 15 from NA19240 (b). All but one *de novo* candidate occurred at a site not found in any of the other samples. This site was PCR tested and identified in NA12892 (mother).
(XLSX)

Table S7 Sub-family breakdown (external Excel file). Fragments from 1,105 of the Alu insertions were assembled into contigs spanning the Alu element to allow subfamily identification. The subfamilies are compared with those from the reference MEI detected as deletions and to the Venter MEI.
(XLSX)

Table S8 Non-reference MEI genotyping validation (external Excel file). Genotype contingency table for non-reference MEI vs. genotypes from PCR validation experiments. “0/0” are homozygous reference, “0/1” are heterozygous insertions, and “1/1” are homozygous insertions (VCF file genotype label convention). Counts in each box are the numbers of sites and samples with the corresponding combination of genotype from sequencing and PCR. The overall genotyping accuracy is the fraction of counts on the diagonal while the genotyping efficiency is the fraction of all genotyped sites & samples divided by sites×samples for the given pilot dataset. Only genotypes with Q≥7 are included. The low coverage (a) accuracy is 87% and the efficiency is 57%. The trio pilot (b) accuracy is 95.7% and the genotyping efficiency is 89.9%. The improved genotyping performance for the trio pilot is a consequence of higher coverage.
(XLSX)

Table S9 MEI genotyping corrections. (external Excel file). a) Detection sensitivity. b) Genotyping efficiency with correction factors used in constructing the allele frequency spectra for each population and element type. c) Heterozygosity counts and correction factors for each sample and element family.
(XLSX)

Table S10 Loss of Function variants (external Excel file). Counts of insertions occurring within genes, UTR, and CDS regions annotated from Gencode version 3b. This table is partially shown as Table 1 in the main text. Only insertions with breakpoint confidence intervals entirely within the annotation region are counted. Any insertion candidate subsequently invalidated is not counted. A random placement model is used to estimate the number of expected insertions in the absence of selection. a) MEI counts. b) The corresponding counts of SNPs from the low coverage pilots are also listed along with the expected numbers of SNPs based on random placement. The suppression factor for MEI (∼46×) is similar to that of a SNP changing a stop codon (∼42×).
(XLSX)

Table S11 Mobile element consensus sequences (external Excel file). Repbase element names and sequences for each of the element added to the reference genome for MEI insertion detection.
(XLSX)

Text S1 The 1000 Genomes Project Consortium.
(DOC)

Text S2 Supporting Methods.
(DOCX)
