# Supplementary figures and images for: A Comprehensive Map of Mobile Element Insertion Polymorphisms in Humans
Source: PLoS Genet. 2011 Aug 18;7(8):e1002236. doi: 10.1371/journal.pgen.1002236 (PMC3158055; doi:10.1371/journal.pgen.1002236)

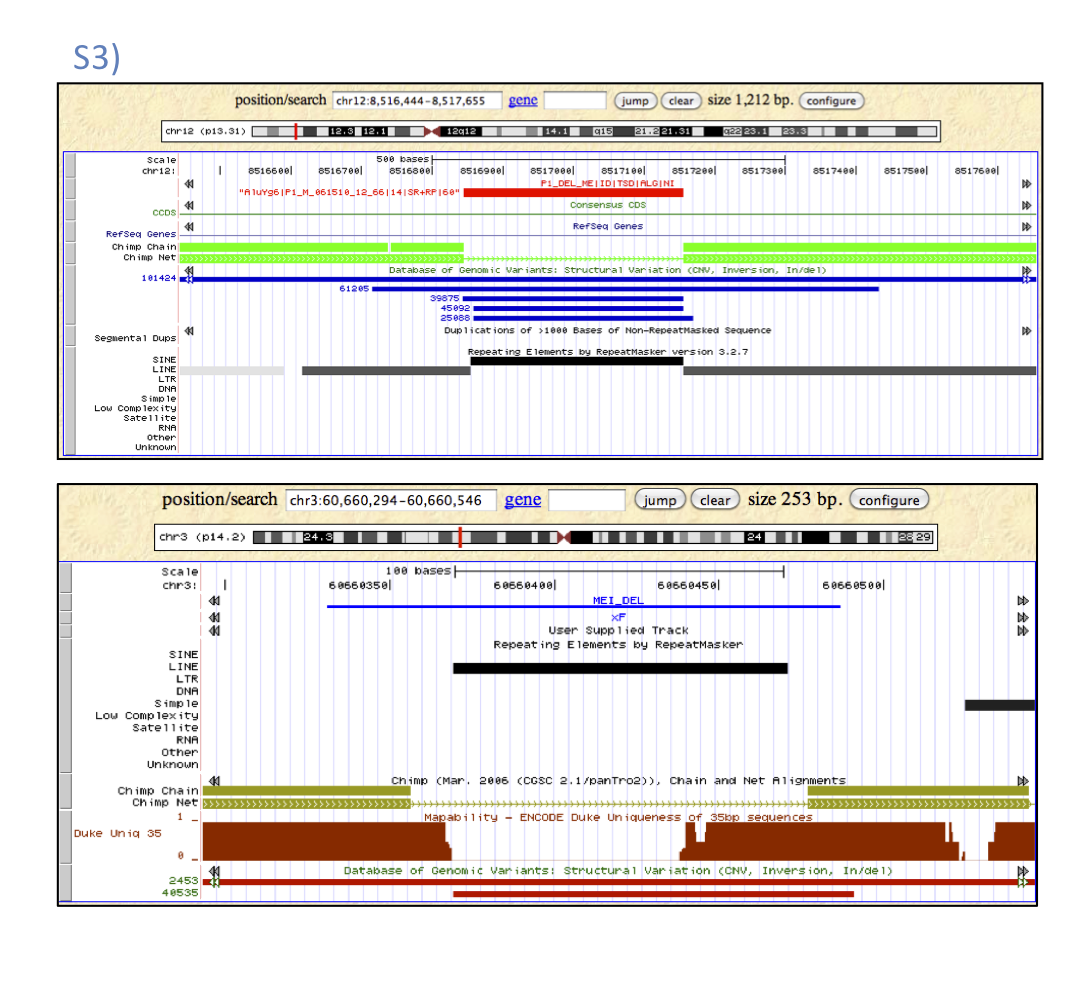

Supplement: Figure S3 — UCSC browser display of reference MEI. (top) The deletion (red track with 1000GP deletion id's P1_M_061510_12_213 for low coverage pilot and P2_M_061510_12_22 from the trio pilot) matches to the annotated AluYg6 element at chr12:8516855–8517156, present in the NCBI36 reference sequence but missing in the sequenced sample. The black RepeatMasker track shows that the AluYg6 element matches the deletion start and end coordinates. The green tracks indicate the extent of the chimpanzee assembly, which does not include the AluYg6 element. The blue DGV tracks show that this particular deletion has been previously identified by several experiments with various degrees of position resolution. (bottom) Example of questionable reference MEI. The blue track at the top marks a detected deletion (id P2_M_061510_3_301) at chromosome 3, 60,660,331 bp that overlaps >50% with a short annotated L1HS element, but the start and end coordinates do not match precisely. The chimpanzee genome (in yellow) has a gap in the region, but the edges do not align precisely. This deletion was included in the count of 2,010 reference MEI, but adds to the level of uncertainty. (TIFF) [file pgen.1002236.s003.tif]

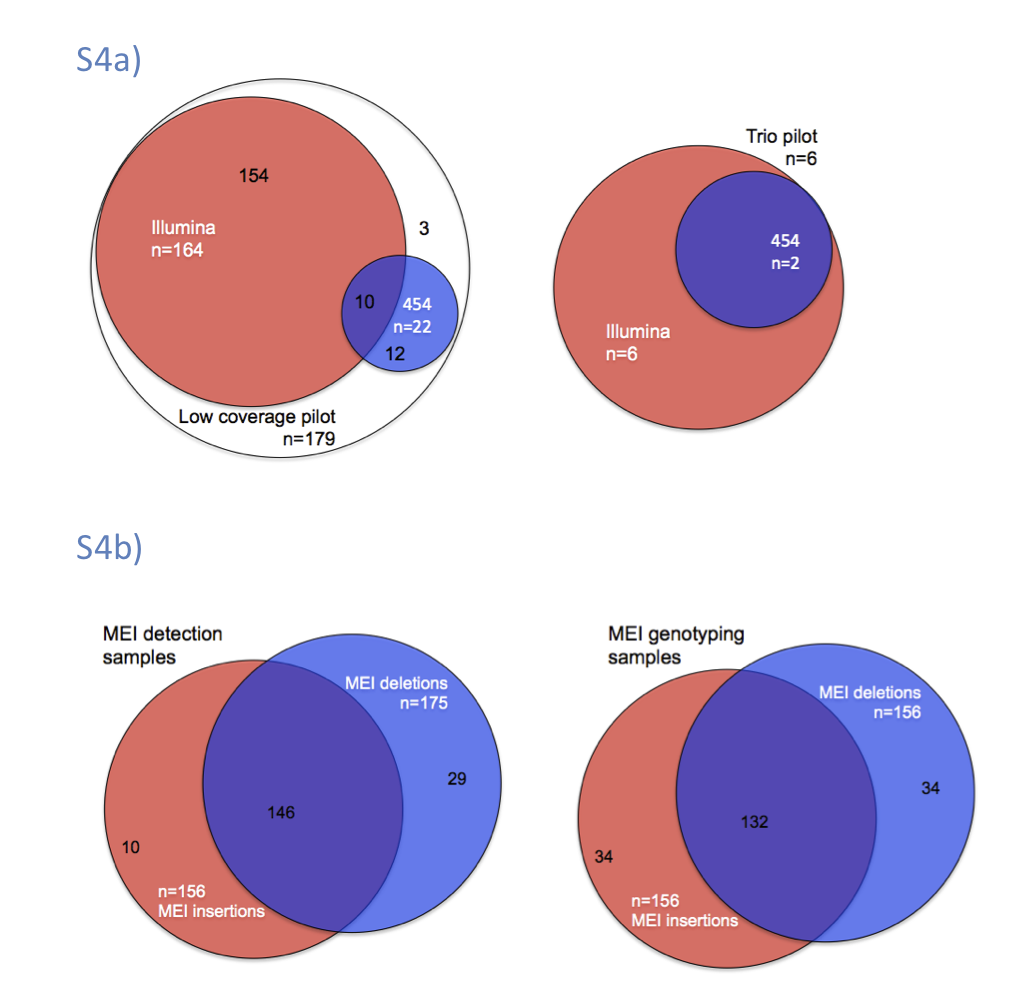

Supplement: Figure S4 — 1000 Genome Project pilot sample breakdown. a) Venn diagram of pilot samples by sequencing platform (Illumina and 454 only). The bulk of the samples were sequenced by Illumina. The circle areas are only roughly proportional to the number of samples contained. b) Venn diagram of samples used for MEI detection (left) and genotyping (right). MEI detected as insertions (red) and deletions (blue) have different signatures and algorithms resulting in the difference between the samples used. (TIFF) [file pgen.1002236.s004.tif]
