# Supplementary material for: A Comprehensive Map of Mobile Element Insertion Polymorphisms in Humans
Source: PLoS Genet. 2011 Aug 18;7(8):e1002236. doi: 10.1371/journal.pgen.1002236 (PMC3158055; doi:10.1371/journal.pgen.1002236)
Supplement: Text S2 — Supporting Methods. (DOCX) [file pgen.1002236.s031.docx]

**Supplemental Information:**

A comprehensive map of mobile element insertion polymorphisms in humans

Chip Stewart^1^, Deniz Kural^1^, Michael P. Strömberg^1^, Jerilyn A. Walker^2^, Miriam K. Konkel^2^, Adrian M. Stütz^3^, Alexander E. Urban^4^, Fabian Grubert^4^, Hugo Y.K. Lam^4^, Wan-Ping Lee^1^, Michele Busby^1^, Amit R. Indap^1^, Erik Garrison^1^, Chad Huff^5^, Jinchuan Xing^5^, Michael P. Snyder^4,^ Lynn B. Jorde^5^, Mark A. Batzer^2^, Jan O. Korbel^3^, Gabor T. Marth^1^, and the 1000 Genomes Project.

*^1^Department of Biology, Boston College, Chestnut Hill, MA*

*^2^Department of Biological Sciences, Louisiana State University, Baton Rouge, LA*

*^3^Genome Biology Unit, European Molecular Biology Laboratory, Heidelberg, Germany*

*^4^Department of Genetics, Stanford University, Stanford, CA*

*^5^Department of Human Genetics, Eccles Institute of Human Genetics, University of Utah, Salt Lake City, UT*

**SUPPLEMENT OVERVIEW**

This document describes the methods and provides additional details about results presented in the main text. Supplementary tables are provided in an external Excel file. The main supplementary table is **Table S1** (external Excel file), which lists each detected event with event properties, such as the element type, inserted sequence when available, and validation status. Many of the results here can be derived from **Table S1**. Another source for information is from the 1000 Genomes Project web site, where MEI event lists have been released in VCF format.

## Data generation.

All whole genome shotgun sequence data was provided by the 1000 Genomes Project [36]. All primary sequence reads, MEI variant calls, inferred genotypes, and validation data are available through the project website ([www.1000genomes.org](http://www.1000genomes.org)). A comprehensive description of the full 1000 Genomes Project pilot data is provided in the supplement to the main pilot paper [36] along with the project web site [www.1000genomes.org](http://www.1000genomes.org). The low coverage pilot and trio pilot datasets were used for MEI detection (i.e. no exon target capture pilot data). Only Illumina read pair data was used for MEI insertion detection by the RP method, and only 454 data was used for MEI insertion detection by the SR method. For MEI detected as deletions, the “release path“ [30] set of deletions was based on all sequence data from both the low coverage and trio pilots. The MEI portion of the released deletion calls were predominantly detected from Illumina read pair data, although evidence from other sequencing platforms was included when the calls were merged into the release. The deletion genotyping pipeline [39] explicitly included information from single end Illumina reads, although this proved to be of little value for MEI genotyping. Individual sample Coriell ID’s, as well as the average span coverage for each sample used by the RP insertion detection method, base coverage for the SR insertion detection method, and the base coverage used in deletion detection are all listed in **Table S2** (external Excel file). Here, “span coverage” is defined as the integrated number of bases in the mapped fragments between the read pairs (not including the mapped parts), since RP algorithms are only sensitive to SVs occurring in the span between read pairs.

## Read mapping.

The MOSAIK program [70] was used to align both the 454 data and Illumina read pair data for non-reference MEI detection. Reference MEI detected as deletions were based on a mixture of several mapping programs used by the 1000 Genomes Structural Variation working group [30]. We mapped all low coverage and trio pilot Illumina and 454 data with MOSAIK [70] version 0.9.1176 using the following alignment parameters: hash size of 15 for Illumina reads with a maximum mismatch of 4 (36mer read length), 6 (51mer read length), and 12 (76 and 101mer read lengths). Parameters for alignment of 454 reads consisted of a hash size of 15 with at least 70 percent of the read being aligned and the maximum percentage of mismatches of 5%. Mapping was performed against the NCBI36.3 version of the human reference genome sequence (like all other 1000GP pilot studies). These sequences were augmented by a mobile element consensus sequence database (termed the “Moblist”) from RepBase [69] sequences for each active mobile element type. The additional sequences included 23 AluY, 6 AluS, 1 SVA, and 22 LINE1 sub-families listed in **Table S11** (external Excel file). For the purpose of MEI detection, MOSAIK prioritized the alignment of reads to the mobile element reference sequences. This was a substantial aid to identify fragments mapping to mobile elements. For each read, MOSAIK reports all possible locations and assigns a quality score to each alignment. This logarithmic phred-like score [51] represents the probability that the read was misaligned. When aligning paired-end reads, MOSAIK performs a local search, defined by a median fragment length and search radius, to rescue the other mate that might otherwise be missed by less sensitive alignment parameters.

Details regarding mapping algorithms for the reference MEI detection are provided in the supplement of the main pilot paper [36]. A breakdown of the mapping algorithms used to detect the subset of deletions identified as reference MEI is listed in **Table S3**. 95% of the MEI sites detected as deletions were found by more than one algorithm but the dominant mapping algorithms were MOSAIK and MAQ [74].

## Insertion coordinate convention

In keeping with the coordinate convention adopted by the 1000 Genomes Project, we use the ‘leftmost’ coordinate convention (i.e. the insertion position is set at the 5’ end of the MEI target site duplication) from the hg18 (NCBI build 36.3) human genome reference. This convention has not generally been adopted by other studies, which adds to the entropy when comparing events between studies. We also report insertion coordinates with estimated 95% confidence intervals, another practice not widely adopted by other studies.

## Limitations of MEI identification

The primary challenge for MEI detection is accurate read mapping because the same read (in the case of the SR method) or the same read pair (in the case of the RP method) must identify the mobile element and the insertion site within the genome. This requires that part of the read (or one mate) maps to a unique locus within the reference genome sequence. This is difficult in the case of MEI events within repetitive regions (e.g. segmental duplications), or hotspots (i.e. regions with multiple mobile elements of the same family in close proximity). Our read-mapping program, MOSAIK [70], was optimized to discriminate uniquely mapped read fragments from those that map ambiguously. Another limitation is the available sequence coverage, particularly in the low coverage samples.

## Non-reference MEI detection.

Read Pair (RP) method. Mobile element insertions are detected by RP as clusters of paired-end fragments in which one end aligns uniquely to the genome and the other end maps to a mobile element reference sequence (main text **Figure 1a**). All "proper-pairs" – read pairs with normal orientation and in which the mapping distance between the pairs is consistent with the library fragment length distribution (p-value>0.005) – are removed from consideration as supporting fragments for candidate MEI insertions since these fragments are consistent with annotated mobile elements. The MEI insertion detection signatures are clusters of fragments that span from the reference coordinates into mobile element sequence in the absence of an annotated element of that family.

SPANNER [40] was used to detect candidate mobile element insertions from the alignment files produced by MOSAIK. Read pairs are classified by mobile element type (Alu, L1, or SVA) and the orientation of the uniquely mapped end. Fragments spanning into the mobile element from the 5' direction are clustered separately from the fragments spanning into the mobile element from the 3' side. With this convention, an insertion can be identified as a two matched clusters of fragments spanning into the insertion from both 5’ and 3’ sides. Duplicate fragments are removed from the clusters, identified as multiple fragments with from the same library with the same mapped coordinates at both ends.

Clustering of supporting fragments is done within the pool of all individuals in a given data set (low coverage & trio pilots were separate pools). The clustering method is a variation on a nearest neighbor algorithm [40,71,72]. The neighborhood distance metric is the genomic distance between centers of fragments. The neighborhood window is determined by the corresponding library fragment length distribution and is set to the span of the library fragment length distribution for any given read. The 0.2% tails of the fragment length distribution are trimmed from the distribution when determining the neighborhood window size. This particular implementation of the nearest neighbor algorithm allows the neighborhood window to adapt to diverse mixtures of libraries with different fragment length distributions. The libraries used here exhibited an assortment of fragment length distributions with means ranging from 100 bp to 500 bp. The flexibility of the clustering algorithm to handle a range of library fragment length scales was an important consideration for pooled insertion detection from the 1000 Genomes Project pilot data set. The compute time needed for this clustering algorithm is linear with the number of fragments considered for clustering because there are no iteration steps in cluster formation. A minimum of two supporting fragments is required in each cluster to ensure that isolated misaligned reads do not lead to false detections. The overwhelming majority of mobile element mapped fragments unambiguously align to one of the three element types (Alu, L1, or SVA) so no sophisticated element family identification logic is required.

Candidate events are formed from clusters spanning into the insertion from both sides within the neighborhood length scale (determined by the fragment length distributions). The RP detection method brackets the position of the insertion somewhere within the gap between the clusters of read-pair ends. This results in an estimate for the insertion position and a corresponding uncertainty in the position.

The estimated position of the insertion is the leftmost edge of the cluster on the 3’ side of the insertion, which corresponds to the 5’ edge of the target site duplication region bracketing the MEI.

The combined effects of read coverage and fragment length limit the insertion breakpoint resolution. The density of read ends in a cluster increases as read coverage increases. The uncertainty of the edge of the cluster is approximated as the average distance between read ends in a clusters.

Following SPANNER processing, the candidate events are filtered in a post-processing step. The candidate MEI are compared with reference coordinates of RepeatMasker annotated [6] loci of Alu, L1, or SVA elements. Event loci that map within 400 bp (approximately the maximum fragment length scale) of a corresponding type mobile element annotation are removed from the list of candidate insertion events. For Alu insertions, only annotated Alu positions are used for masking. For L1 and SVA candidates, the corresponding annotations for L1 and SVA positions are augmented by Alu positions. This masking step compensates for residual alignment inefficiencies for finding proper pair alignments in the neighborhood of mobile elements. False detections arising from misalignment artifacts in low complexity repeats and variable number tandem repeats (VNTR) are removed by masking within a 30 bp window around annotated VNTR and simple repeats, also downloaded from the UCSC table browser [5].

In addition to annotation masking, selection criteria filter out low confidence insertions. Clusters are rejected if the uniquely mapped ends of the reads coincide with regions enriched in multiple mapped reads. Candidate masking and quality filtering leave a sample of candidate events highly enriched in true insertions.

Simulations. SPANNER was optimized and tested on samples of simulated paired-end data with Alu and L1 insertions. The simulation was based on the hg18 chromosome 20 sequence with 1000 AluY and 1000 L1HS full-length elements inserted randomly as heterozygous insertions. No inserted element was allowed within 100 bp of an annotated element. Paired-end reads were generated from the diploid genome using the ART read simulation program [73] which introduced base calling errors randomly according to a typical base quality distribution from Illumina 35 bp reads. 32M reads were generated (~18x coverage) with fragment lengths of 150 bp, 200 bp, and 500 bp in equal amounts. The detection efficiency for Alu insertions was 83%, and for L1 insertions 81%, with a false detection rate less than 1 per 63 Mb (no false detections on chromosome 20). Prior to masking and post-processing event selection, the detection efficiency exceeded 99%, but with 118 false detections in chromosome 20 (extrapolating to several thousand false detections from the full genome). This level of post-processed detection performance from these simulations gave us confidence in the approach when applied to real data. The SR method was also tested on the same simulated data. The SR detection efficiency exceeded 95% without the need for additional filter criteria.

Non-reference MEI detection using the 454 split-read (SR) method (main text **Figure 1b**). MOSAIK processed all 454 reads from the 1000 Genomes low coverage and trio pilot data. The output contained the mapped locations in MOSAIK read archive format as well as FASTA sequence files for all reads for which there was no mapped location meeting the alignment criteria (at least 70 percent of the read being aligned with a maximum percentage of mismatches of 5%). The set of all unaligned reads was then re-aligned allowing for partial read mapping to the mobile element consensus sequences using MOSAIK. The following alignment criteria were used: 15 bp hash size, alignments with more than 5% of mismatch bases were filtered out, and at least 40 bp of the read must align to one of the mobile element consensus sequences. For each re-aligned read, a target region was created from alignments to the mobile elements. The target region is defined as the largest spanning region where multiple overlapping alignments hit. The read was discarded if multiple, non-overlapping alignments were found. The remaining sections before and after the target region are compared. The longer section is kept for further processing, while the other section of the read is trimmed away. If less than 40 bp remained after trimming, the read was discarded. Trimmed reads were then aligned to the hg18 reference. The longest alignment was selected if it was at least 5 bp longer than the next longest alignment, otherwise the read was discarded. The remaining reads were considered candidate fragments that support an MEI event. The candidates were checked for novelty by aligning the entire read back to the hg18 reference using relaxed alignment parameters (up to 9% of the read bases could be a mismatch and the aligned length could vary from 90 – 100% of the read length). All candidates that were aligned using the relaxed parameters were discarded from the detection pipeline.

The split-read mapping allows up to three unaligned regions in each candidate fragment: a gap occurring before the genomic hit (genome gap), after the mobile element hit (mobile element gap), and between the genomic and mobile element hits (mid gap). Candidates with a mid gap longer than 6 bp were discarded. Candidates with a genome or mobile element gap longer than 6 bp were also discarded, except when the target region contained the entire mobile element. Candidates were discarded if any of the following criteria were true: the alignment quality score was less than 40, the mobile element alignment length was less than 60 bp, candidate occurred within 100 bp of a RepeatMasker annotation of a family Alus, L1s, and SVAs corresponding to the insertion type.

## Reference MEI selection.

The various deletion detection methods employed by the 1000 Genomes Structural Variation working group are described elsewhere [30,36,38-44]. The full deletion event list was formed from high confidence and validated calls from 23 different 1000 Genomes deletion call sets. Selection of the reference MEI subset from the full release set of deletions (n=22025) from 1000 Genomes Project pilot data [30,36] was based on matching the deletion coordinates to RepeatMasker 3.27 [6] Alu, L1, and SVA annotations, with the additional requirement that the ME element not be present in the chimpanzee genome [37] (6x *pan Trogodytes*-2.1 assembly) at the corresponding position (main text **Figure 1d**). The matching to mobile element annotation required both ends of the deletion to be within 50bp (200bp) of the mobile element ends for Alus, L1s, or SVAs. Reference MEI were then selected from the matched calls based on having no more than 25% percent of a deleted region covered by assembled chimpanzee genome lifted to hg18 coordinates. The hg18 and the chimpanzee (PanTro2) assembly were downloaded from the UCSC table browser as a bed file showing the extent of contigs and clones comprising the chimpanzee Build 2, Version 1 (Oct. 2005).

Following this selection filtering, 2010 deletions were identified as MEI that inserted in the reference sequence and were polymorphic within the sequenced samples. MEI sites detected as deletions were detected by 16 of the 23 different call sets, predominantly Illumina RP and SR detection algorithms (**Table S3**). Multiple algorithms detected the vast majority of sites.

A typical reference MEI is shown from the UCSC browser in **Figure S3** with precise coordinate matching of the detected deletion with the annotated element and a gap in the chimpanzee assembly. The bottom panel is a rare example of a questionable reference MEI due imprecise coordinate matching. Both events are among the 2,010 reference MEI.

## Samples

The breakdown of samples by pilot, sequencing platform, detection method, and genotyping method, and population group is a potentially confusing topic. All in all the 1000 Genomes Pilot included 179 samples low coverage pilot data and 6 samples (two family trios) in trio pilot data (totaling 185 sequenced genomes) but not all samples were sequenced by each technology. **Table 1** (main text) lists the numbers of samples for each different MEI detection method. A breakdown of samples is provided in **Table S2**, which lists Illumina paired-end span-coverage, 454 base-coverage, and Illumina single-end plus paired-end base-coverage for all samples in the 1000 Genomes low coverage and trio pilot data, even samples that were not sequenced by Illumina or 454 technology (three samples had AB SOLiD data only). Illumina paired-end span-coverage is the relevant coverage metric for the RP insertion and deletion detection, 454 base coverage for SR detection methods. The number of samples for the Illumina data far exceeded the 454 data (**Figure S4a**). The number of samples used for non-reference and reference MEI genotyping were balanced at 156 each (**Figure S4b**) but overlapped in only 132 samples. The 23 different deletion call sets from the 1000 Genomes Structural Variation subgroup used nearly all possible samples (182 of 185) but the deletion genotyping was limited to Illumina data only.

## Validation methods

Gel band scoring rules. Each agarose gel contained two lanes of DNA ladder, one 100 bp (cat. No. 170-8352) and one 500 bp (cat. No.170-8354) EZload^TM^ molecular ruler (BioRad Laboratories, Inc. Hercules, CA). An amplicon matching the size predicted for the pre-insertion site was scored as a zero (0) while the insertion-present amplicon was scored as a one (1). Individuals homozygous absent were scored as 0,0, homozygous present as 1,1 and heterozygotes were scored as 1,0 for each allele and recorded in an Excel spreadsheet for further analysis. **Table S4** (external Excel file) is the matrix of PCR genotypes for each DNA sample at tested MEI sites.

Observed band sizes were estimated using the available size markers and recorded together with the number of bands visible. Afterwards, they were compared to the sizes of the reference and the predicted insertion allele and a call if normal, heterozygous or homozygous insertion was made. Results from two enzymes (iProof High Fidelity DNA Polymerase (Biorad) and Hotstart Taq (Qiagen) ) were compared. If they did not agree, a follow-up PCR test was performed until a clear decision could be made, or until the experiment was judged inconclusive. Note that the iProof enzyme and conditions were more likely to give a signal for longer PCR fragments due to its higher speed but that the HotStart Taq showed less background signal and thus was overall more specific.

Preliminary PCR experiments. In a preliminary round of PCR validation experiments, LSU designed PCR primers for 183 MEI detection events (135 Alu and 48 L1) with an overall FDR of 13.1% (24/183). In this dataset, the FDR for Alu was 4.4% (6/135) and for L1 was 37.5% (18/48). These preliminary results were used to further optimize algorithm parameters at BC. **Table S4a** lists these loci with validation status and the laboratory at which the experiments were carried out. The additional PCR validation experiments were designed and carried out for preliminary testing, validation for exon-interrupting insertions, and for testing the *de novo* MEI candidate site.

## Non-reference MEI detection sensitivity

RP and SR sensitivities depend on respective sequence coverage. The RP method is sensitive to sequence “span coverage” defined as the number of bases covered by the fragments between the reads on the ends of the fragments. Only SV breakpoints occurring within the un-sequenced middle of the fragment can be detected by an RP method. On the other hand “base coverage” governs the detection sensitivity of SR methods since the SV breakpoint can occur anywhere in the read (except close to the edges of the read). Each trio child had different span and base coverage, which result in different levels of detection sensitivity. This is shown in **Figure S6** where the detection sensitivity for each method in both samples is plotted against coverage (Illumina read pair span coverage on the bottom for RP detection, and 454 base coverage on the top for SR detection). The dependence of sensitivity on coverage was estimated by down-sampling the number of supporting fragments linearly with coverage, counting the fraction of candidate MEI insertions that pass selection criteria.

The RP method shows a saturation at ~70% detection above span coverage of ~10x fragments/base per sample. This reflects an upper limit to the RP method detection sensitivity arising from the MEI candidate filter step masking out all positions within 400 bp of an annotated mobile element. This masking window size was set by the scale of the Illumina RP fragment length distributions to eliminate the possibility that the end mapping into a mobile element does not correspond to an annotated mobile element. This wide masking window for the RP method resulted in the ~30% drop in detection sensitivity. The SR method did not need to impose such a severe masking criterion, so the SR detection sensitivity continues to rise beyond 70% at high coverage.

Non-reference MEI detection also depends on the inserted element family. The breakdown by element family for overlapping detection between the two methods (independent datasets) in the trio children samples can be used to estimate sensitivity in exactly the same way as was done in the main text (**Methods: Detection sensitivity**). **Table S6** shows the counts as well as the sensitivity estimates for each family and each detection method. Statistical errors on the L1 and SVA sensitivities are relatively large, so for purposes of keeping simplicity we apply detection sensitivity values listed in **Table S9** for low coverage analysis.

## Non-reference MEI position resolution

We quantified the insertion position resolution of the RP and SR methods by comparison with each other and comparison to dbRIP insertion positions. A matching window of 100 bp was used to select 1793 loci found by both RP and SR methods. The position residual between matched RP to SR insertions (**Figure S7**) has a sharp peak at 0, indicating that both algorithms approach single base resolution with sufficient read coverage around the insertion. Tails extend out to 25 bp and the standard deviation of the position residuals is 9 bp.

Matching of the 1000GP non-reference MEI list to dbRIP was done with a 200 bp window by which we selected 632 MEI loci. The dbRIP coordinates appear to have been reported mostly using the ‘rightmost’ convention so the dbRIP coordinates were moved 1 target site duplication (also listed in dbRIP) to the left so that both lists share the ‘leftmost’ convention. The standard deviation of the residual is 20 bp.

## Comparisons with previous studies

MEI insertion positions from dbRIP [29] and nine other recent publications [23-28,34,35,45] were compared with the event list to check for overlap. Detected insertions within a window of 200bp of previously known insertions were labeled with an event ID from the previous study in **Table S1**. In cases where the previous study identified the insertion within wide bracketing coordinates (eg. primer coordinates), the average of the coordinates was used to estimate the insertion position.

Overlap of MEI deletion loci from previous studies. MEI sites detected as deletions were compared with known deletion coordinates from dbVar (www.ncbi.nlm.nih.gov/dbvar, June 2010) and the huRef MEI deletion coordinates [23]. Based on the deletion start and end coordinates, any MEI deletion with 50% reciprocal overlap with a previously discovered deletion was labeled with the study ID in **Table S1**.

The two recent studies [34,35] used comparable detection methods and identified many non-reference MEI loci. The low overlap of our detected loci with these two studies (**Figure S8**) is partially explained by incomplete overlapping samples in the datasets, however there are other differences.

Many insertions identified only in those studies, not included among the 5370 insertions of this study, are located within close proximity to annotated elements of the same family (**Figure S9**), regions which this study avoided due to expected false detections arising from the inability to distinguish variant inserted elements from annotated elements within the range of the fragment length. This is a limitation of the RP method, but not particularly for SR. Distance to the closest element of the same family should be expected have roughly the same distribution for non-reference and reference MEI, and this is generally what is observed in this study and from huRef [23]. The spikes at 1 bp in **Figure S9** are non-reference MEI loci detected inside annotated elements of the same family. When matched to dbRIP non-reference MEI, the position residuals from the two recent studies have relatively wide distributions (**Figure S10**). Some of the difference can be explained by the added breakpoint resolution of SR methods.

## MEI Hotspot

Despite limited sensitivity to regions of MEI pileup, we found evidence for an MEI hotspot in the vicinity of the HLA region (**Figure S11**). The counts of MEI loci per 1Mb bin should be Poisson distributed with an average density of λ ~ (5370)/(2.85Gb), which does fit the measured distribution well, except for the HLA region. The hotspot is also apparent on the main text **Figure 2a** as a spike in chromosome 6.

## Insertion length estimate.

For the majority of non-reference MEI loci without a full assembly, the insertion length is estimated as the span of mapping coordinates within the inserted element. The accuracy of this estimate depends on several assumptions, prime among them are that the insertion consists of the inserted element identified by the “moblist” sequence matching most of the fragments in the cluster and the insertion contains very little else (e.g. transduced sequence or long poly-A tails). The accuracy can be assessed by comparison with matching our MEI insertions to fully assembled insertions from dbRIP [29]. The insertion length scatter-plot and resolution distribution are shown in **Figure S12.** The resulting insertion lengths from non-reference MEI are compared to the insertions length distribution from reference MEI detected as deletions in **Figure S12c.** All-in-all, assembly is the superior way to measure insertion length.

## Genotyping Performance

Genotyping performance is quantified terms of efficiency (percent of sites x samples genotyped at the detected sites) and accuracy (genotype quality, GQ, as a proxy for accuracy). Non-reference and reference MEI have very diferent trade-offs between accuracy and efficiency. There is a steep drop in efficiency with increasing GQ threshold (**Figure S13**, top panel, blue line) for non-reference MEI, but less of a drop in efficiency for reference MEI (red line). The stepping in blue for on-reference MEI correspond to increasing numbers of supporting fragments used to call the genotype and the GQ value (**methods: Eqn. 5 and 6**) where GQ≥7 corresponds to 2 or more supporting fragments. No such stepping is apparent in the reference MEI GQ cumulative distribution because of the additional evidence (read depth and LD with local SNPs) used by GenomeSTRiP. The shaded region is the 95% confidence interval for genotype agreement with PCR genotypes at a given GQ threshold. From this we selected GQ≥7 for non-reference MEI where the genotyping efficiency is 42% of detected in the low coverage data. Reference MEI sites with GQ≥10 corresponds to 80% genotyping efficiency. Genotyping efficiency improves with increased sample read coverage (**Figure S13**, bottom), particularly for non-reference MEI.

Genotyping performance can also be assessed by the rate of Mendelian errors in the family trios and by the consistency of called genotypes with Hardy-Weinberg Equilibrium (HWE). Among the 2531 MEI insertion sites in the trio pilot samples, there were three sites with Mendelian errors in the YRI trio, and seven sites with errors in the CEU trio. The proportion of genotypes for each population is consistent with the expected dependence on allele frequency according to Hardy-Weinberg Equilibrium (**Figure S14**).

## Number of ME polymorphisms between pairs of individuals

The number of pair-wise variant sites in the main text **Figure 6b** between two samples was calculated as the total difference of insertion sites between samples. We account for MEI detected as insertions as well as deletions and use the respective detection sensitivities and false detection rates (FDR) to make a best estimate of the true number of variant loci (**Table S6**). The number of pair-wise variations is counted as the symmetric difference between MEI detected in the two samples:

|  | $\Delta N =(N1+N2)\cdot\frac{(1-FDR)}{\varepsilon}-2\cdot\frac{N12}{\varepsilon^{2}}$ | (S1) |
| --- | --- | --- |

where N1 and N2 are the counts of MEI events detected in each sample; N12 is the count of MEI detected in both samples; the MEI detection sensitivity is ε, the efficiency to detect the MEI in both samples is then ε^2^. This is calculated separately for MEI deletions and insertions, with the respective FDR and ε values and then added together for the total number of pair-wise MEI differences. Uncertainties in the ΔN estimates are derived from the Poisson errors in N1, N2, and N12, and the uncertainties in the detection FDR and sensitivity by error propagation:

|  | $\begin{matrix} \sigma_{\Delta N}^{2} =\left(\frac{\partial\Delta N }{\partial N1}\right)^{2}\cdot N1+\left(\frac{\partial\Delta N }{\partial N2}\right)^{2}\cdot N2 \\ \begin{matrix} & +\left(\frac{\partial\Delta N }{\partial N12}\right)^{2}\cdot N12+\left(\frac{\partial\Delta N }{\partial FDR}\right)^{2}\cdot\sigma_{FDR}^{2}++\left(\frac{\partial\Delta N }{\partial\varepsilon}\right)^{2}\cdot\sigma_{\varepsilon}^{2} \end{matrix} \end{matrix}$ | (S2) |
| --- | --- | --- |

Values of ΔN with uncertainties are listed in **Table S6** for pairs of samples from the trio pilot data and displayed as error bars in **Figure 6d**. The pairing of NA12878 and NA19240 (trio children) has the highest accuracy because both Illumina and 454 high coverage data was available for the trio children, while the parents were sequenced only with Illumina technology. Detection sensitivities, false detection rates (FDR), and corresponding uncertainties (σ_ε_, σ_FDR_) are taken from **Table S6** for non-reference MEI, and **Table S3** for reference MEI detected as deletions. Counting pair-wise differences (ΔN) from the low coverage samples suffers from significantly greater uncertainty arising from the lower detection sensitivity (ε) and uncertainties in the sensitivities (σ_ε_) for each sample.

## Principal Component Analysis.

We carried out a Principal Component Analysis (PCA) of the MEI genotypes to assess patterns for MEI within population groups. For each site with at least 100 samples genotypes meeting the quality criteria (GQ≥7 MEI insertions and GQ≥10 for MEI deletions) a matrix of insertion allele count for each low coverage sample (rows) and site (columns) was constructed. The elements of the matrix (**Figure S15**) have values of 2 (homozygous MEI), 1 (heterozygous MEI), or 0 (no MEI). Elements of the matrix without genotypes were set to the average allele count for all genotyped sites and samples.

We used the Matlab *princomp* function (Matlab version R2010a) to calculate the first and second principal components which are plotted in **Figure S16** for four datasets. Starting with genotypes from the PCR validation samples and sites, we expected that the MEI patterns would cluster more tightly with population group as information is added to the input allele count matrix.

## Modeling the MEI allele Frequency Spectrum

The observed MEI allele frequency spectrum (combined non-reference + reference) is compared to a θ/i neutral theory model [52-54]. The θ parameter is functionally equivalent to the nucleotide diversity parameter from SNP models and *i* is the count of insertion alleles in a sample of *N* individuals (2N chromosomes). This represents the expected allele frequency of a new allele introduced to a population of constant size. Components of the MEI allele frequency spectrum, non-reference (detected as insertions) and reference (detected as deletions) are separately not consistent with the θ/i model because the detection introduces a strong ascertainment bias in the allele frequency, namely that the MEI must either be present in the reference (MEI deletions detected as the absence of the insertion in a given sample) or must not be present in the reference (MEI insertion detected as an insertion in a given sample). A simple model for the ascertainment bias can be made with the assumption that the reference represents one more sampled chromosome added to sequenced samples in which an MEI may occur. Given an MEI allele of frequency *AFS(i)=i/2N*, the probability that the insertion will appear in the reference is *P_REF_=i/2N.* The reference MEI allele frequency at count *i* becomes:

|  | $\begin{matrix} AFS(i)_{REF}=(\theta/i)\cdot P_{REF} \\ \begin{matrix} & & = \end{matrix}(\theta/i)\cdot(i/2N)=(\theta/2N) \end{matrix}$ | (S3) |
| --- | --- | --- |

a flat spectrum. The probability that the insertion is not in the reference is just *(1-P_REF_ )* so the non-reference MEI *AFS(i)* is:

|  | $\begin{matrix} AFS(i)_{NREF}=(\theta/i)\cdot(1-P_{REF}) \\ \begin{matrix} & & = \end{matrix}(\theta/i)\cdot(2N-i)/2N \end{matrix}$ | (S4) |
| --- | --- | --- |

which is a spectrum that falls steeper than *(*θ*/i*) to zero as AF approaches 1.

## Coalescent Models.

We estimated the coalescent time between YRI and CEU trio individuals using the re-sequencing data from one 100 kb ENCODE region (ENr123) obtained from the ENCODE 3 project (Main text **Figure 6d**). For the point estimate, we used the phylogenetic method described by Thomson et al [60]. Briefly, the number of derived alleles from each pair of individuals (*S*) was counted in this region, and the expected coalescent time *E(T)*, between the pair of individual is calculated by *E(T)=S/(4*µ*L)*, where *µ i*s the per-bp, per-year mutation rate (5.9*10^-10^) and *L* is the length of the region (100 kb). The per-year mutation rate for this region was estimated based on the human-chimpanzee divergence in this region (1.2%) using the method described in Nachman & Crowell, 2000 [61], with a generation time of 25 years, a human-chimpanzee speciation time of six million years ago, and a human-chimpanzee ancestral effective population size of 84,000 (averaged from the estimates from [62-64]. We then bootstrapped the SNP dataset 1000 times on each pair of individuals to obtain the 95% confidence intervals. Because the sequence was only available for the trio parents, we estimated the coalescent time between the two children (NA12878 and NA19240) by averaging all pair-wise comparison between the parents. The resulting coalescent time estimates for the three pairs of individuals are:

| CT(NA12878,NA19240) = 978.4 (783.6-1175.5) ky |
| --- |
| CT(NA19238,NA19239) = 711.6 (571.8-861.9) ky |
| CT(NA12891,NA12892) = 542.2 (413.0-690.4) ky |

We also modeled the expected MEI allele frequency spectrum with a coalescent (**Figure S17**) to explore the effects of population bottlenecks, migration, and population subdivision. As we would expect, there is a substantial increase in rare variants relative to the random mating model due to bottlenecks and population subdivision. This produces a noticeable skew towards rare variants in all three spectra. In addition, there is a relatively minor increase in the proportion of high-frequency derived states, due to the interaction of population subdivision and subsequent population bottlenecks. This increase is difficult to see in the unbiased and MEI detected as insertion (non-reference) spectra, but it produces a substantial skew towards high-frequency variants in the MEI detected as deletion (reference) spectrum, which may, at least in part, explain why we observe more high-frequency reference MEI spectrum. The coalescent simulations implemented the best-fitting model of Yoruban-European-East Asian population history from Schaffner, 2005 [55] with sample sizes of 66 CEU individual, 48 YRI, and 52 CHB/JPT. We estimated the unconditional allele frequency spectrum by summing over the length of each branch in each genealogy, and normalizing by the total branch length of all 100,000 genealogies.

## Stability of population specific mutation rates

The estimated MEI mutation rates depend on choices for the selection of genotyped sites; the more selective the quality criteria, the larger the corrections to compensate for lost genotypes. If the corrections are stable, the MEI heterozygosities will be consistent as the selection criteria change, but the statistical errors will increase as fewer sites pass the selection criteria. GQ≥7 corresponds to the loosed possible criteria sensitive to heterozygous sites, but we can also demand more fragments per genotype call to increase genotyping accuracy at the cost of decreased statistics. Such a scan over the selection criteria (**Figure S18**) shows that the MEI insertion rates are stable and that the pattern of population specific insertion rates remains as the number of fragments supporting the genotype is increased from 2 to 7.

## 
